# Integration of DNA methylation and gene expression analysis in *Gephyrocapsa huxleyi* provides insight into genes related to calcification

**DOI:** 10.1093/g3journal/jkag076

**Published:** 2026-05-20

**Authors:** Xiaoyu Zhang, Betsy Read

**Affiliations:** Department of Computer Science and Engineering, California State University San Marcos, San Marcos, CA 92078, United States; Department of Biological Sciences, California State University San Marcos, San Marcos, CA 92078, United States

**Keywords:** *Gephyrocapsa huxleyi*, DNA methylation, epigenetic regulation, gene expression, calcification, biomineralization, coccolithophore, functional genomics

## Abstract

Coccolithophores, such as *Gephyrocapsa huxleyi* (formerly *Emiliania huxleyi)* play a key role in oceanic carbon cycling through calcification. However, the molecular mechanisms regulating coccolith formation remain unclear. In this study, we performed an integrative analysis of whole-genome bisulfite sequencing (WGBS) and RNA-Seq data on two closely related strains of *G. huxleyi*: the calcifying M217 and the non-calcifying CCMP1516. We identified over 17,000 differentially methylated regions (DMRs) and more than 12,000 differentially expressed genes (DEGs) between the two strains.

Our findings reveal statistical associations between DNA methylation patterns—particularly in CpG contexts—and changes in gene expression, implicating epigenetic regulation of calcification. Functional enrichment analyses highlighted genes involved in dynein complex activity, phosphate transport, carbonic anhydrase function, and calcium signaling as key contributors to biomineralization. Statistical modeling confirmed that hypo-methylation in promoter and gene regions is positively associated with gene expression, especially in calcification-related genes. However, the expression differences between the strains are complex and cannot be fully explained by methylation changes alone. This work provides new molecular insights into epigenetic regulation of biomineralization in coccolithophores, with implications for understanding environmental adaptation and biogeochemical cycling.

## Introduction

Coccolithophores play a crucial role in oceanic carbon cycling through the calcification of coccoliths and photosynthesis. The intricate structures of the coccoliths have attracted significant attention from researchers across various fields, including material science, geology, biogeography, ecophysiology, and, notably, the biomedical community, due to potential applications in bone formation and related diseases (Abedin et al. 2004; Giachelli 2005). Biomineralized tissues, such as bones and teeth, are of fundamental importance in medicine and healthcare. However, the mechanisms governing the formation of these tissues are not yet fully understood. Investigating the design principles of coccolith structures could provide insights with potential applications in material science, such as the development of novel materials for periodontal structures, bone scaffolding, biomedical implants, and membranes for high-temperature separations (Gordon and Parkinson 2005).

While many coccolithophores have restricted distributions, *Gephyrocapsa huxleyi* (*G. huxleyi*), formerly *Emiliania huxleyi*, stands out due to its exceptional breadth of distribution, making it the dominant bloom-forming coccolithophorid and one of the most ubiquitous and abundant species of oceanic phytoplankton. *G. huxleyi* has become a model organism for studying biosphere-geosphere interactions and is the subject of extensive physiological, biochemical, and ecological research (Gordon and Parkinson 2005; Read et al. 2013). The sequencing and annotation of its genome (Read et al. 2013) have made *G. huxleyi* an ideal model system for studying biomineralization in coccolithophores.

Coccoliths in *G. huxleyi* are synthesized through a matrix-mediated process (Brownlee et al. 2015; Dedman et al. 2024). The nature, orientation, size, and shape of the crystalline elements are controlled within a specialized compartment adjacent to the nucleus, known as the coccolith vesicle (Outka and Williams 1971). A Golgi-derived reticular body attaches to the coccolith vesicle, and is hypothesized to deliver calcium and key matrix macromolecules, including proteins, polysaccharides, proteolipids, and proteoglycans (Klaveness 1972; Sviben et al. 2016; Beuvier et al. 2019). Once the coccolith is fully formed, the reticular body disintegrates, and the vesicle migrates to the cell surface, where the coccolith is extruded in a massive exocytotic event (Paasche 1998; Marsh 2003). This process results in the formation of an interlocking sphere of platelets that encapsulate the cell. Cellular proteins are thought to coordinate biomineralization and coccolithogenesis, playing roles in ion transport, crystal nucleation, growth, and patterning, as well as vesicle trafficking and exocytosis (Dedman et al. 2024).

DNA methylation is a heritable epigenetic mark in many eukaryotic organisms and plays a crucial role in regulating genome structure and transcription (Phillips 2008; Suzuki and Bird 2008; Huff and Zilberman 2014; Dhar et al. 2021). It is an essential epigenetic modification that controls gene expression during normal cellular differentiation, development, disease pathogenesis, and aging (Smith and Meissner 2013; Jin and Liu 2018). Hypermethylation of CpG sites or islands near the transcription start site (TSS) is often associated with the repression of gene transcription (Feng et al. 2010; Zemach et al. 2010). However, recent studies have shown that the relationship between DNA methylation and gene expression is much more complex. Binary classifications of hyper- or hypo-methylation in promoter regions have often revealed only weak negative correlations with gene expression levels (VanderKraats et al. 2013; Wagner et al. 2014; Schultz et al. 2015; Li et al. 2020). Moreover, while DNA methylation was once thought to be relatively stable, it is now recognized as being both enduring and reversible, and hence key to epigenetic plasticity.

Two closely related strains of *G. huxleyi*, the calcifying M217 and the non-calcifying CCMP1516, provide an ideal model system for studying the effects of epigenetic modifications on gene expression and biomineralization regulation. Strain CCMP1516, isolated from the South Pacific in 1991 (02.6667S, 82.767W), was maintained at the Bigelow National Center for Marine Algae and Microbiota, where it lost its ability to calcify over time. At the time of isolation, a subclone was also sent to the Plymouth Algal Collection and designated M217 (M217). While M217 retained its ability to calcify, the two strains have likely similar genomes, as evidenced by the 100% identity of the nucleotide sequences in *cox3*, *tufA*, and the mitochondrial and plastid 16S rRNA (Supplementary File 1). Whole-genome DNA methylation has not been systematically studied for *G. huxleyi* or other coccolithophores. Given the similar genomes of these strains, methylation is hypothesized to play a key role in the phenotypic differences in calcification between CCMP1516 and M217.

To investigate the relationship between DNA methylation and gene expression in M217 and CCMP1516, we generated RNA-Seq and whole-genome bisulfite sequencing (WGBS) libraries for six samples (three biological replicates per strain). The two strains exhibited significantly different profiles in both gene expression and DNA methylation. We analyzed the DMCs and DMRs in the genome and used a Bayesian linear regression model to relate the log2 fold changes (LFCs) in gene expression to the mean methylation levels of DMRs and specific genomic regions, including promoter, 5′ UTRs, and gene bodies.

Furthermore, we showed that there are statistical correlations between differential gene expression and the number of DMCs in gene regions. However, the expression differences between the strains are complex and cannot be fully explained by methylation changes alone. We also performed comparative studies of differential gene expressions in calcifying *G. huxleyi* strain under growth conditions promoting calcification vs control. Enrichment analysis of differentially expressed genes (DEGs) revealed several interesting genes and biological processes in *G. huxleyi* related to biomineralization.

## Materials and methods

### Sample preparations and data generation

*G. huxleyi* strain CCMP1516 was obtained from the Bigelow National Center for Marine Algae and Microbiota and M217 from the Plymouth Algal Collection (Plymouth, UK). Both strains are subclones of the same sample isolated from the South Pacific (02.6667S 82.767W) and considered to be isogeneic. The originally calcifying CCMP1516 lost its ability to calcify over time, but M217 retained its ability to calcify. Cultures were grown at 17–19 °C in 0.2 μm filtered artificial seawater (ESAW, Berges et al. 2001) with metals and vitamins added to achieve f/2 medium concentrations (Guillard and Ryther 1962). The medium contained 200 μM nitrate and 40 μM phosphate, and cultures were grown under a 12-h-light/12-h-dark cycle with cool white fluorescent light at 660 μmol m^−2^ s^−2^. Nucleic acids were extracted from three biological replicates for each strain during the same mid to late log phase of growth and at the same time (7 d post inoculation), in the middle of the light phase (11:00–12:00 Am). Batch cultures were split such that RNA was extracted using a standard guanidinium isothiocyanate procedure (Strommer et al. 1993), while genomic DNA was isolated using the cetyltrimethylammonium bromide (CTAB) method and further purified using a cesium chloride density gradient (Wilson 2001). Nucleic acid concentrations were measured by SYBR Green fluorescence, purity assessed via *A*_260_/*A*_280_ and *A*_260_/*A*_230_ absorbance ratios, and integrity evaluated by agarose gel electrophoresis. High quality samples were sent to Beijing Genomics Institute (BGI) for RNA-Seq and bisulfite library preparation and sequencing. Bisulfite library preparation and sequencing was performed using standard well-validated protocols using the ZYMO EX DNA Methylation Gold Kit. Bisulfite conversion efficiency was calculated using spiked-in unmethylated control DNA: Conversion efficiency = 1-number of unconverted C/Total C in control, and was determined to be ≥99%.

After sequencing on the Illumina HiSeq 2500 platform (2 × 150 paired-ended reads), adapters and low-quality reads were filtered from the raw data by BGI. Supplementary Tables 1 and 2 present the statistics of the RNA-seq and WGBS libraries, respectively. Each of the six RNA-seq libraries contained 49–54 million clean reads with a length of 100 bp, representing more than 50-fold coverage of the *G. huxleyi* transcriptome. Each WGBS library generated 38.1–41.6 million clean reads with a length of 150 bp, providing approximately 34- to 37-fold coverage of the *G. huxleyi* reference genome (Read et al. 2013).

### Differential expression analysis

RNA-seq reads were mapped to the masked diploid *G. huxleyi* CCMP1516 reference genome (Read et al. 2013) using STAR (Dobin et al. 2013) with default parameters, and gene-level counts were generated using the summarizeOverlaps function with gene exons in the GenomicAlignments R package (Lawrence et al. 2013). Differentially expressed genes (DEGs) were identified by taking the union of results from the DESeq2 (Love et al. 2014) and edgeR (Robinson et al. 2010) R packages, using digital gene counts. A false discovery rate (FDR) < 0.05 was applied as the threshold for determining DEGs.

KEGG pathway enrichment analysis of the DEGs was performed using KOBAS (Xie et al. 2011), while GO enrichment analysis was conducted using Trinotate-based GO annotations and tools (Bryant et al. 2017), with all *G. huxleyi* genes in the JGI filtered best model serving as the background. The enriched GO terms were visualized using REVIGO (Supek et al. 2011).

### Differential methylation analysis

The *G. huxleyi* reference genome has a high GC content of 65.7% and contains nearly 18 million CpG sites. Bismark (v0.18.0) (Krueger and Andrews 2011) was used to map the WGBS libraries to the *G. huxleyi* genome and extract methylation calls for individual cytosines using default settings. The standard Bismark tools “bismark2report” and “bismark_methylation_extractor” were then used to extract methylation info at each cytosine site in the genome and to generate mapping report and strain level statistics. Table 1 shows the mapping statistics of the bisulfite sequences. The WGBS libraries represent biological replicates, not paired across strains. WGBS library names are not correlated with the RNA-seq library names. The sample “CCMP1516A” was excluded from further analyses due to its anomalously low GC content (31%, compared to 34% in the other samples) and low mapping efficiency (19.1%), likely caused by contamination or sequencing errors. The remaining five samples showed mapping efficiencies ranging from 45.3% to 52.3%, with a mean rate of 49.74%, and achieved genome coverage above 73%, indicating high data reliability and accuracy.

**Table 1.** Mapping statistics of bisulfite sequences to the *G. huxleyi* genome (the sample CCMP1516 A was excluded from the analysis because of its low mapping efficiency of 19.1%).

Sample Name	Mapping efficiency	Genome coverage	Average depth	Bisulfite conversion ratio	CpG methylation	CHG methylation	CHH methylation
CCMP1516A	19.1%	41.6%	6.93	97.9%	9.0%	4.8%	0.5%
CCMP1516B	48.2%	73.9%	18.09	97.9%	15.88%	5.43%	0.5%
CCMP1516C	45.3%	73.3%	15.59	98.1%	15.74%	5.20%	0.4%
M217A	51.7%	74.1%	18.74	97.6%	15.51%	6.08%	0.6%
M217B	52.3%	74.9%	19.63	97.7%	15.50%	5.98%	0.6%
M217C	51.2%	73.7%	19.25	97.7%	15.40%	5.98%	0.5%

A paired *t*-test was performed on the average CpG and CHG methylation levels across the 100 longest scaffolds of the *G. huxleyi* genome. The null hypothesis (H_0_) was that the true mean difference is zero, indicating no systematic difference in methylation profiles between the strains. For CpG methylation, the mean difference across the 100 scaffolds was 0.33% (with CCMP1516 showing slightly higher methylation on average), and the standard deviation of the differences was 0.19%, yielding a *t*-statistic of 16.9 (df = 99) and a *P*-value < 2.2e−16. For CHG methylation, the mean difference was −0.58% (with M217 showing higher methylation on average), and the standard deviation of the differences was approximately 0.19%, yielding a *t*-statistic of −29.9 (df = 99) and a *P*-value < 2.2e−16.

Let $N_{i,l}^{s}$ represent the coverage of CpG site *l* in the *i*th sample of strain *s* (s=1for CCMP1516 and s=2 for M217). The Bismark mapping results revealed 11,966,862 CpG sites with $N_{i,l}^{s}\;\geq 3$ across all samples of both strains, and 8,219,026 sites with $N_{i,l}^{s}\;\geq 10$. For the subsequent differential methylation analysis, we considered only those sites with $N_{i,l}^{s}\;\geq 3$ in all samples. Let $\beta_{l}^{s}$ denote the methylation level of strain *s* at the *l*th CpG site. As shown in Supplementary Fig. 4, the histogram of *β* displayed two peaks near 0 and 100%. Using thresholds β<10% for unmethylated sites and β>90% for fully methylated sites, we identified 109,847 CpG sites that were unmethylated in one strain but fully methylated in the other. Among these, 38,871 were fully methylated in M217 and 70,976 in CCMP1516. Thus, CCMP1516 contained significantly more fully methylated CpG sites than M217, which may affect its gene expression. Pairwise Spearman correlations of methylation levels in the CpG and CHG contexts were also calculated across samples. Correlations within the same strain were significantly higher than those between strains, indicating that the two strains have distinct methylation profiles.

We investigated different methods for identifying DMCs between strains M217 and CCMP1516. Let $C_{i,l}^{s}$ denote the number of methylated cytosines at the *l*th CpG site in sample *i*. First, we modeled the count $C_{i,l}^{s}$ using a GLM with binomial distribution:


$$C_{i,\;l}^{s}∼\mathrm{Binom}(N_{i,\;l}^{s},\;\beta_{l}^{s}),$$

where $\mathrm{logit}(\beta_{l}^{s})=\alpha_{l}^{s}$, with a Gaussian prior $\alpha_{l}^{s}\;∼\;\mathrm{Norm}(0,\;1.5)$. We then computed posterior distributions of $\beta_{l}^{s}$ using Bayesian updates and mapping data at site *l* across all samples, which incorporated mapping depth and sample variation, and produced confidence intervals for the estimates. DMCs were defined as the sites with a 99% confidence interval and posterior mean difference $|{\hat{\beta }}_{l}^{1}-{\hat{\beta }}_{l}^{2}|>25\text{\%}$. This approach identified 313,073 CpG DMCs, of which 189,988 were hypo-methylated in M217 $\left(\beta_{l}^{1}>\beta_{l}^{2}\right)$ and 123,085 were hyper-methylated in M217 $\left(\beta_{l}^{1}<\beta_{l}^{2}\right)$. Using the methylKit package (Akalin et al. 2012), which employs the beta-binomial model from DSS package (Park and Wu, 2016), we identified 263,293 DMCs under the threshold of $|\beta_{l}^{1}-\beta_{l}^{2}|>25\text{\%}$ and *q*-value < 0.01. Similarly, MOABS (Sun et al. 2014), which applies a beta-binomial hierarchical model to increase statistical power, identified 283,833 CpG DMCs using the Bismark mapping data and default parameters, including 173,528 hypo-methylated and 110,305 hyper-methylated in M217. The results across methods were highly consistent: 99.6% of methylKit DMCs were also found in the MOABS set, and over 99.99% of MOABS DMCs overlapped with those from the GLM. For subsequent analyses, we selected the MOABS DMCs, balancing statistical power with false positive control.

Because of the large numbers and widespread distribution of DMCs, many studies focus on identifying differentially methylated regions (DMRs), which represent clusters of DMCs. In practice, DMRs are defined as genomic segments containing a minimum number of nearby DMCs (e.g. at least three within a distance threshold). However, different software packages often rely on ad hoc parameter choices and produce divergent DMR sets, such as DSS (Park and Wu, 2016) and MOABS. For example, DSS identifies 12,053 CpG DMRs with an average length of 22.2 bp, whereas MOABS identifies 12,123 DMRs with a much longer average length of 56.2 bp.

We found that different tools often produced highly inconsistent DMR sets; for instance, 4,181 (34.7%) of DSS DMRs and 5,052 (39.5%) of MOABS DMRs do not overlap with any DMRs in the other set. Manual inspection indicated that the MOABS results were the most consistent and sensitive. For example, the large DMR in the MOBAS result shown in Supplementary Fig. 9 was not detected in the DSS DMR set. Therefore, we used the DMC and DMR results generated by MOABS for further analysis.

### Correlation between differential methylations and gene expressions

To avoid potential biases arising from selecting a particular set of DMRs, we directly estimated the effects of CpG methylation variation on gene expression. To do this, we constructed generalized linear models (GLMs) to capture the relationship between gene expression log fold changes (LFC) and relevant DMCs. Because of the complexity of these relationships and the many other factors influencing gene expression, our goal was not to predict expression levels from methylation alone but rather to identify statistically significant effects of differential methylation on gene expression.

The statistical model incorporated the number of DMCs in specific regions associated with genes, such as gene bodies, promoters, and upstream regions from the TSS. The expression level of gene g of sample i, ie, the number of reads mapped to the gene, is modeled using a negative binomial (gamma-Poisson) distribution:


$$E_{g}^{i}∼NB(\lambda_{g}^{i},\phi ),$$

where the exponential distribution scale factor ϕ∼Exp(1). We applied a GLM for the mean $\lambda_{g}^{i}$ such that


$$\log\;\lambda_{g}^{i}=\log\;\lambda_{m}+e_{g}+f_{i}+\beta_{+}\cdot s\cdot X_{g}^{+}+\beta_{-}\cdot s\cdot X_{g}^{-}.$$

-
$\log\;\lambda_{m}\;$ is the logarithm of the global median expression level.
-
$e_{g}$ ∼ Normal(0,3) represents variation for individual gene *g*, with a large variance in the prior to account for the wide range of gene expression levels.
-
$f_{i}\;∼$ Normal(0, 0.1) accounts for sample variations.
- The strain variable s=0 for CCMP1516 and s=1 for M217.

We considered only genes containing DMCs in the feature region of interest (e.g. gene bodies or promoters). Let $DMC_{g}^{+}$ and $DMC_{g}^{-}$ denote the number of hyper-methylated (higher in M217) and hypo-methylated (lower in M217) DMCs in the feature region of gene *g*, respectively. Then


$$X_{g}^{+}\;=\;\log(DMC_{g}^{+}+1),\;X_{g}^{-}\;=\;\log(DMC_{g}^{-}+1)$$

normalized to the range [0, 1]. The coefficients $\beta_{+}$ and $\beta_{-}$ model the effect of hyper- and hypo-methylated sites on gene expressions, with Gaussian prior Normal(0, 1.5).

Posterior distributions of the random variables were estimated using Hamiltonian Monte Carlo (HMC) implemented in the RStan package (Stan Development Team 2025). Non-zero values of $\beta_{+}$ and $\beta_{-}$ with high confidence indicate significant effects of differential methylation on gene expression. The coefficients $\beta_{+}$ and $\beta_{-}$ were separately estimated for CpG and CHG DMCs, as well as different feature regions (gene regions or promoters).

## Results

### Genome-wide patterns of DNA methylation in *G. huxleyi*

#### Overall methylation levels

The average methylation level provides a general overview of the methylome. It is calculated as the ratio of reads with methylated cytosines to the total number of mapped reads covering the site. Cytosine methylation levels vary depending on the local sequence context (C, CpG, CHG, and CHH) as well as the strains of *G. huxleyi*. While the two *G. huxleyi* strains had similar overall cytosine methylation levels close to 7.1%, the average methylation level in the CpG context was higher in CCMP1516 (approximately 15.8%) compared to M217 (approximately 15.5%), with a significant *P*-value < 2.2e–16 using the Wilcoxon signed rank test. In contrast, M217 had a higher average methylation level (approximately 6%) in the CHG compared to CCMP1516 (approximately 5.3%), with a significant *P*-value < 2.2e–16 (Table 1). CHH sites showed negligible methylation levels across scaffolds and were not analyzed further.

Supplementary Fig. 2 shows the average methylation levels across the 100 longest scaffolds in the *G. huxleyi* genome differ consistently between the strains, although the absolute differences are relatively small. In the CpG context, mean CpG methylation levels per scaffold varied significantly, ranging from 11.53 to 18.93% in CCMP1516, with an average of 15.81%, and from 11.33 to 18.40% in M217, with an average of 15.48%. The differences between the two strains were statistically significant (*P* < 2.2e–16, paired *t*-test), with CCMP1516 showing higher CpG methylation levels in 97 out of the 100 scaffolds.

In the CHG context, M217 consistently exhibited higher methylation levels across all scaffolds, ranging from 5.04 to 7.27%, with a mean of 6.15%, compared with CCMP1516, which ranged from 4.29 to 6.49%, with a mean of 5.44%. These differences were also statistically significant (*P* < 2.2e–16, paired *t*-test).

**Fig. 1.**
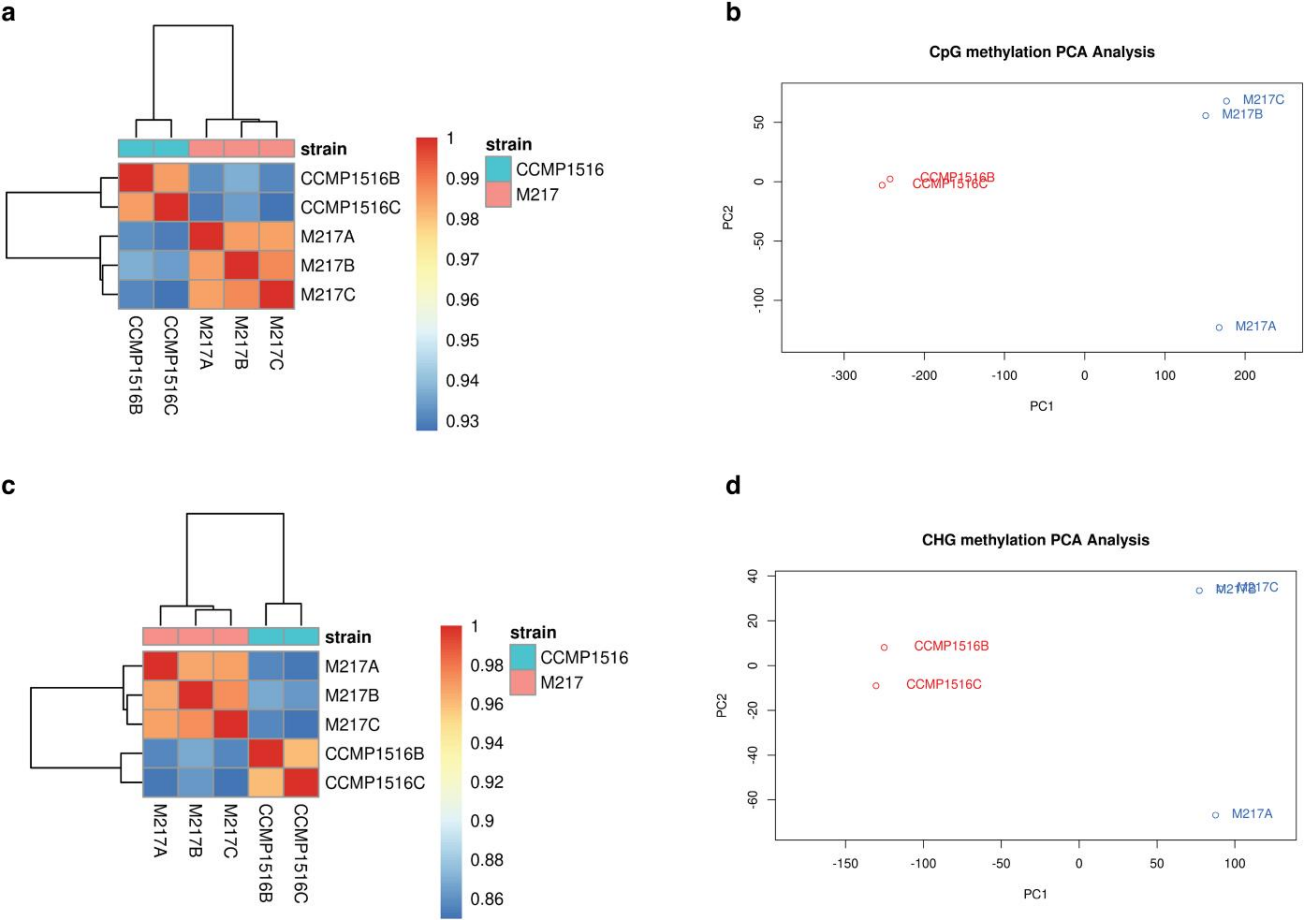
(a) Correlation heat maps and (b) principal component analysis plots of *G. huxleyi* methylation profiles in the CpG (c) and CHG (d) context show replicates for individual strains are highly correlated but different across strains indicating the data is robust. Only cytosine sites with coverage depth ≥10 in all samples are used in the analysis.

Despite the similar overall methylation levels, the two strains exhibited distinguishable methylation profiles across the genome. Figure 1 illustrates the correlations and PCA plots of methylation profiles for the two strains in the CpG and CHG contexts, considering only cytosine sites with coverage ≥10 in all samples. The samples from the same strain exhibited a significantly higher average correlation than those from different strains in both the CpG and CHG contexts. In the CpG context, the average correlation between samples of the same strain was 0.986, compared with 0.932 across strains. In the CHG context, the mean correlation was 0.967 within strains and 0.857 across strains. The PCA plots further support that the two strains exhibit distinct genome-wide methylation patterns, which may contribute to differences in gene expression profiles and physiological traits.

An overview of genomic methylation levels in 10 kbp windows on scaffolds 1 and 14, as shown in Supplementary Fig. 3, reveals significant variation in methylation rates across the *G. huxleyi* genome. Scaffold 1, the longest scaffold, exhibited similar methylation profiles between the two strains, with a correlation of 0.966 in the CpG context, and 0.935 in the CHG context. In contrast, scaffold 14 exhibited a greater difference in methylation profiles, with a correlation of 0.943 in the CpG context and 0.885 in the CHG context between CCMP1516 and M217.

Supplementary Fig. 4 illustrates that most cytosines in the *G. huxleyi* genomes are unmethylated, with more than 80% of CpG sites and over 90% of CHG sites showing methylation levels below 10%. Among cytosines with methylation level >10%, the highest methylation levels (90–100%) were most prominent, which included ∼12% of CpG sites and ∼3% of CHG sites.

#### Methylation in different genomic regions

**Fig. 2.**
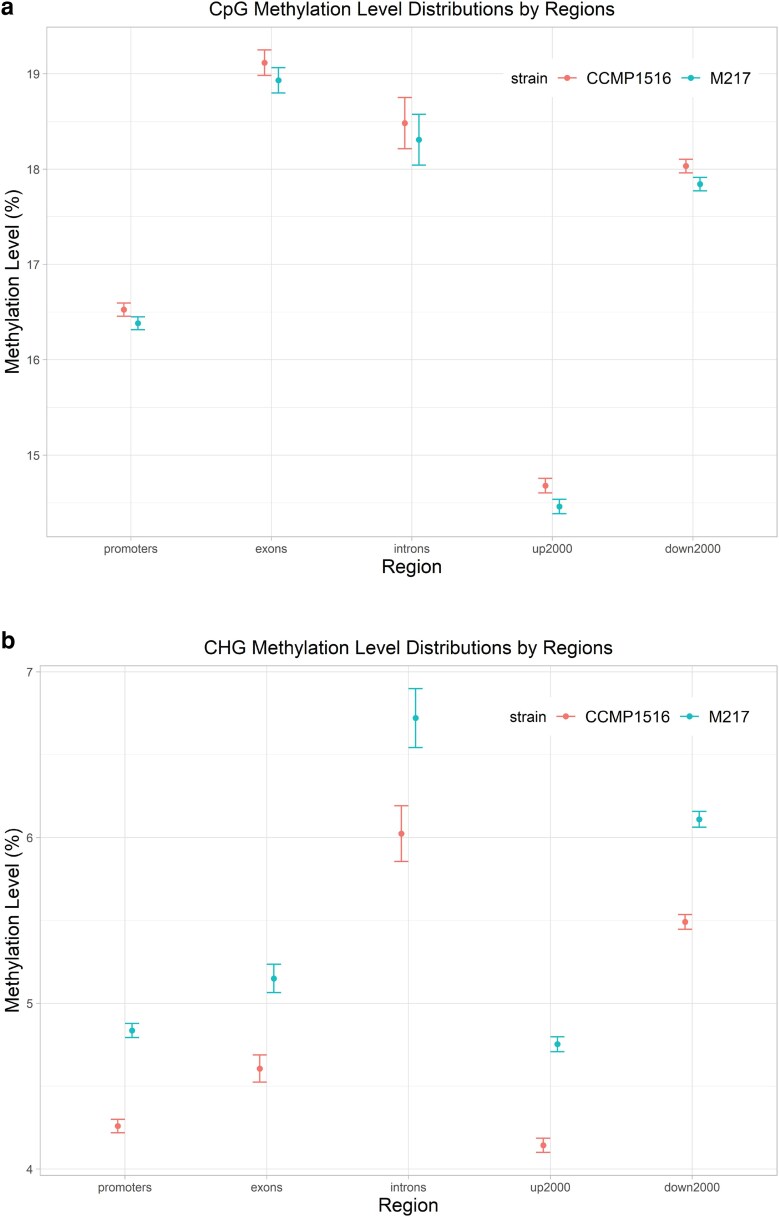
Strains CCMP1516 and M217 exhibit distinct region-specific methylation patterns for both CpG (a) and CHG sites (b), with M217 generally displaying higher methylation levels across most regions. The error bars showed the 95% confidence interval around the mean methylation level.

To compare DNA methylation patterns across different regions of the *G. huxleyi* genome, we computed the average methylation levels in various regions surrounding annotated genes (Fig. 2). With a focus on regulatory methylation patterns as opposed to strict architecture, promoter regions were defined as 1,000 bp upstream and 1,000 bp downstream from the transcription start site (TSS) (Tang et al. 2017), while “up2000” and “down2000” represent regulatory regions upstream and downstream of the TSS, respectively.

In the CpG context, exons exhibited the highest average methylation levels, whereas the “up2000” regions had the lowest. Apart from the introns, significant differences in CpG methylation levels were observed between the CCMP1516 and M217 strains in the up2000 (*P* ∼ 6e−10), down2000 (*P* ∼ 2e−10), and promoter regions (*P* ∼ 5e−6), as well as in the exons (*P* < 0.004). Notably, CCMP1516 displayed higher average CpG methylation levels across all these regions.

In contrast, CHG methylation levels were significantly higher (*P* < 2.2e−16) in M217 than in CCMP1516 across all genomic regions. Introns showed the highest CHG methylation levels, while the up2000 region exhibited the lowest.

These analyses revealed significant differences in methylation patterns between the CCMP1516 and M217 strains, particularly in the up2000, down2000, and promoter regions. The methylation variations observed in these functional elements may explain some of the differences in gene expression and phenotypical traits between the two strains.

### Differential methylation analysis

In this study, we identified significant differences in levels of differentially methylated cytosines (DMCs) between the two *G. huxleyi* strains using MOABS (version 1.3.4). A total of 303,106 DMCs were detected in the CpG context, averaging 1.83 DMCs per 1000 bp and representing approximately 2.2% of all methylated CpG sites. Of these, 185,182 were hypo-methylated and 117,924 were hyper-methylated in strain M217 compared to CCMP1516. We hypothesize that the greater number of hyper-methylated DMCs in CCMP1516 may contribute to reduced expression of associated genes, particularly those that may be involved in calcification including the “glutamic acid-proline-alanine” (GPA) glycoprotein, carbonic anhydrases, ion transporters, cytoskeletal proteins, and calcium binding proteins.

In the CHG context, we identified 200,872 DMCs, averaging 1.21 per 1,000 bp and accounting for about 1.69% of all methylated CHG sites. Among these, 135,246 were hyper-methylated and 65,626 were hypo-methylated in M217 relative to CCMP1516. This disparity reflects the significantly higher mean CHG methylation levels observed in M217.

Supplementary Fig. 5 illustrates the distribution of DMCs across scaffolds. In the 100 longest *G. huxleyi* scaffolds, the average number of CpG DMCs per 1,000 bp ranged from 0.98 to 8.45 (mean: 2.2, SD: 1.3), while CHG DMCs ranged from 0.8 to 3.68 (mean: 1.42, SD: 0.41). The average DMC densities in the 100 longest scaffolds were significantly higher than those in the rest of genome. Longer scaffolds contain more DNA sequence, and hence more CpG sites, and potential regulatory regions; all of which increases the chance of detecting methylation changes. A strong correlation was observed between the frequencies of CpG and CHG DMCs, with a Pearson correlation coefficient of 0.85. Genomic regions with more CpG changes also tend to have more CHG changes, suggesting coordinated regulation of methylation contexts.

**Fig. 3.**
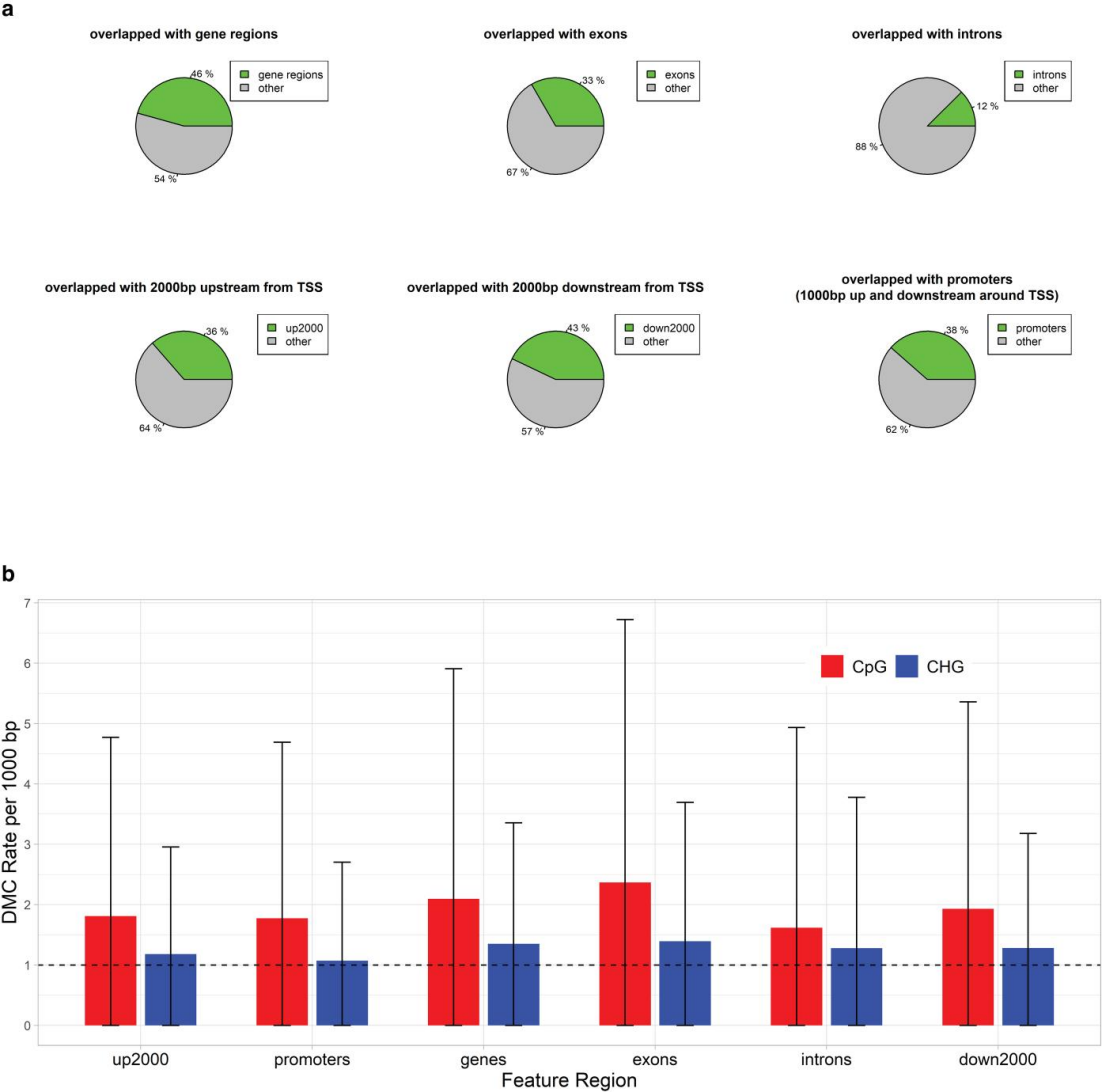
The percentage of CpG differentially methylated cytosines (DMCs) overlapping various feature regions (a), and the average rate of DMCs per 1,000 bp in these regions (b) presents a complex methylation landscape. While introns contain the smallest total percentage of CpG DMCs, exons show the highest density per unit length.

Figure 3 illustrates the distribution of DMCs relative to various genomic features of *G. huxleyi*. DMCs were more concentrated in annotated gene regions, which constitute approximately 39.8% of the genome. These regions contained 46% of the CpG DMCs (33% in exons, 12% in introns) and 44% of the CHG DMCs (30% in exons, 15% in introns, as shown in Supplementary Fig. 10). The frequency of CpG DMCs per 1,000 bp was highest in gene regions, particularly exons, with rates of 2.1 and 2.37 in the CpG context. Similarly, in the CHG context, gene regions and exons had elevated DMC rates of 1.35 and 1.39, respectively. By contrast, introns and promoters exhibited fewer DMCs per 1,000 bp.

Differentially methylated regions (DMRs), defined as contiguous stretches of DNA with multiple cytosine sites showing consistent methylation changes, provide greater biological insight than isolated DMCs. In this study, we identified 12,123 DMRs in the CpG context, ranging in size from 6 to 2,243 bp (average length: 56 bp). In the CHG context, 5,062 DMRs were detected, with an average length of 43 bp.

Differentially methylated genes (DMGs) are genes that overlap with DMRs. We identified 3,780 DMGs, which intersected with 49% of the 17,185 DMRs in the combined CpG and CHG contexts. In the CpG context, 52.3% of DMRs overlapped with 2,481 unique genes, representing 6.5% of all annotated *G. huxleyi* genes. In the CHG context, 40.3% of DMRs overlapped with 1,748 unique genes.

Differential methylation in promoter regions upstream from gene TSS often affects gene transcription. Differentially methylated promoters (DMPs) are defined as 2,000 bp segments upstream of TSS overlapping DMRs. We identified 4,693 differentially methylated promoters (DMPs) in both CpG and CHG contexts, representing43.7% of all DMRs.

**Fig. 4.**
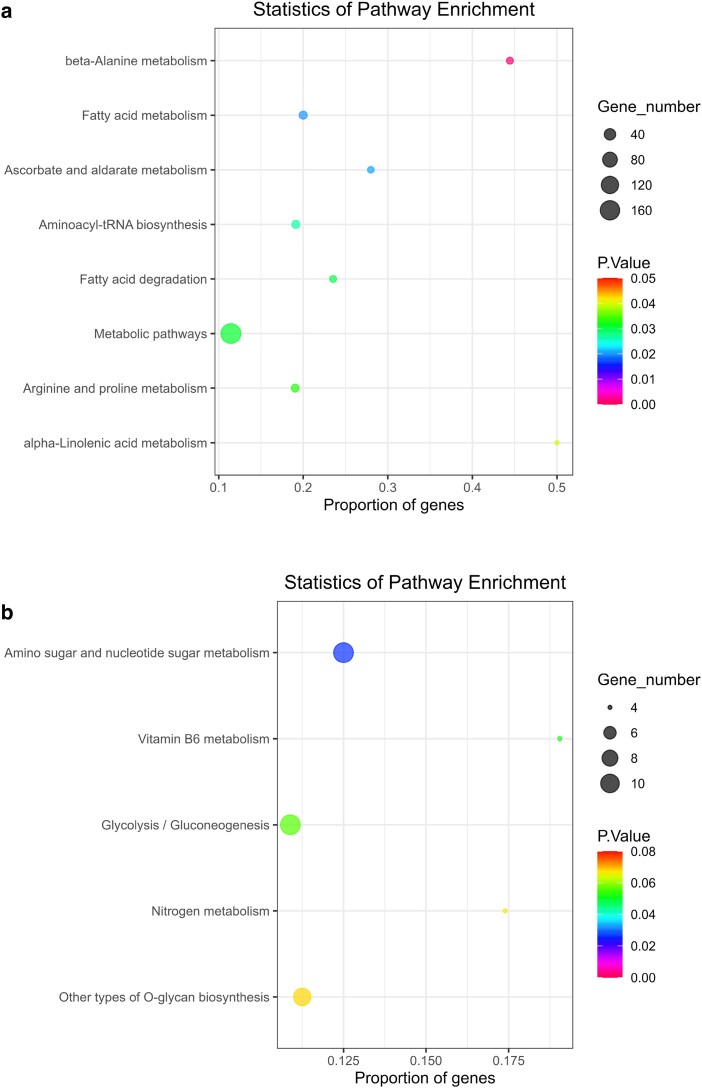
KEGG pathway enrichment analysis shows that differential DNA methylation associated with gene upregulation impacts distinct sets of biological pathways depending on whether the methylation change occurs within the gene body (a) or in the promoter region (b). Gene body methylation changes are broadly associated with amino acid and lipid metabolic pathways and protein synthesis; whereas promoter methylation changes are linked to carbohydrate metabolism and cofactor synthesis. The *x* axis reflects the proportion of differentially methylated (DM) genes in each pathway, the number DM genes is indicated by the area of the circle (a), and the color represents the corrected *P*-value range (b). Note that genes in different KEGG categories may overlap.

**Fig. 5.**
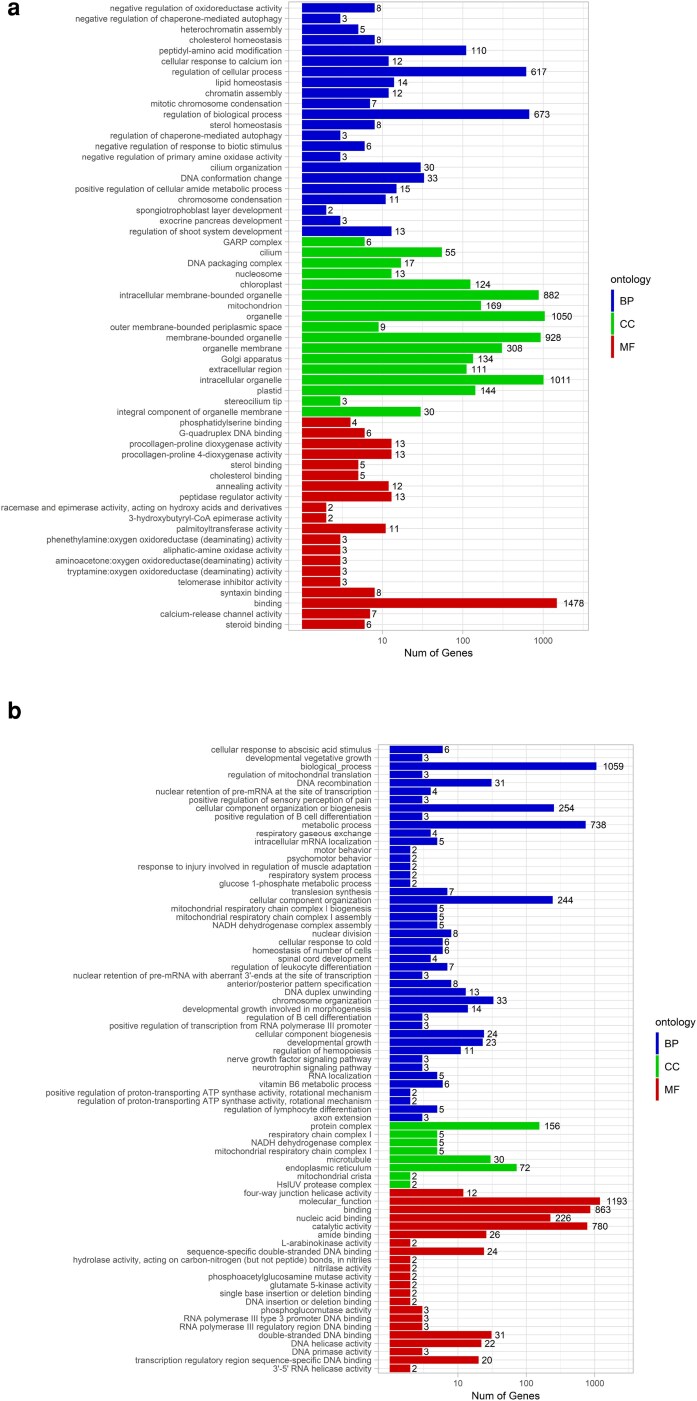
Top enriched GO terms (*P* < 0.01) in biological process (BP), cellular component (CC), and molecular function (MF) identified by enrichment analysis of DMGs (a) and DMPs (b). The numbers of differentially methylated genes annotated with each GO term were shown above the bars. GO enrichment analysis suggests that differential DNA methylation of genes (DMGs) and promoters (DMPs) serve as key epigenetic modulators of various biochemical processes, metabolic pathways, and cellular functions.

Enriched Kyoto Encyclopedia of Genes and Genomes (KEGG) pathway analysis using KOBAS 3.0 (Xie et al. 2011; Bu et al. 2021) revealed that DMGs are associated with beta-alanine metabolism and fatty acid metabolism, while DMPs are linked to amino sugar metabolism and vitamin B6 metabolism (Fig. 4). Gene Ontology (GO) enrichment analysis further shows that DMGs are enriched in biological processes such as inhibition of oxidoreductase activity (*P* = 6.4e−4), peptidyl-amino acid modification (*P* = 2.9e−3), lipid homeostasis (*P* = 3.6e−3), chromatin assembly (*P* = 5.4e−3), cilium organization (*P* = 6.6e−3), and DNA conformation changes (*P* = 6.8e−3), as shown in Fig. 5 and Supplementary Fig. 6. DMGs enriched in cellular components, include the Golgi-associated retrograde protein (GARP) complex (*P* = 8.7e−6), cilium (*P* = 2.5e−4), DNA packaging complex (*P* = 2.6e−4), and the Golgi apparatus (*P* = 4.9e−3). Enrichment in chromatin assembly and DNA packing is consistent with methylation driven changes in genome structure. These enrichment results also suggest methylation may be key to regulation of calcification in *G. huxleyi, g*iven that calcification in coccolithophores occurs in a Golgi-derived intracellular vesicle and relies on the transport of ions and matrix molecules from the Golgi apparatus (Mackinder 2012). The molecular functions of enriched DMPs are associated with binding (*P* = 3.9e−4) and four-way junction helicase activity (*P* = 1.1e−4), as well as biological processes such as cellular response to abscisic acid stimulus (*P* = 4.1e−4) and developmental vegetative growth (*P* = 8.2e−4).

### Differentially expressed genes (DEGs) between *G. huxleyi* strains

We performed differential expression (DE) analysis on RNA-seq data of the two *G. huxleyi* strains, with three biological replicates per strain. Count matrices were generated by aligning the RNA-seq reads to the *G. huxleyi* reference genome (Read et al. 2013). The RNA-seq libraries showed high mapping efficiencies, ranging from 82% to 94% (Supplementary Table 3). Differential gene expression analysis identified 12,711 genes with significant DE between strains M217 and CCMP1516, at an FDR threshold of 0.05. Among these, 6,234 genes are overexpressed in M217 relative to CCMP1516, while 6,477 genes are under-expressed. This DE gene set provides a valuable resource for exploring the relationship between DNA methylation and gene expression in *G. huxleyi*.

Despite M217 and CCMP1516 being closely related strains, significant differences in gene expression levels were observed across the samples, leading to notable phenotypic variations. Supplementary Fig. 7 illustrates the correlations in the gene expression profiles between the two strains across all six samples. The average correlation within the M217 samples was 0.977, and within the CCMP1516 samples was 0.944, both of which were significantly higher than the mean correlation across strains (0.806). In Supplementary Fig. 7b, the PCA plot shows clear separation of the two strains along the first principal axis of the normalized gene expression profiles.

**Fig. 6.**
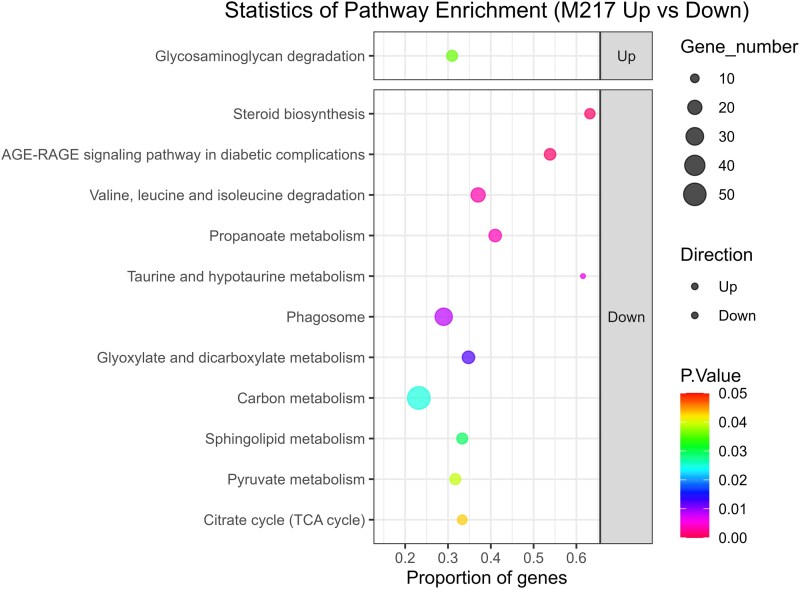
KEGG pathway enrichment analysis of DEGs shows that *G. huxleyi* strain M217 exhibits a distinct metabolic shift compared to 1,516, characterized by upregulation of glycosaminoglycan degradation and significant downregulation of metabolic processes including steroid biosynthesis, amino acid metabolism, carbohydrate metabolism (TCA cycle, pyruvate, carbon), lipid metabolism and cellular functions such as phagosome activity. The *x* axis reflects the proportion of DEGs in each pathway; the number of DEGs is indicated by the circle area; and the color represents the corrected *P*-value.

The KEGG pathway analysis identified the “Glycosaminoglycan degradation” pathway as significantly enriched among the up-regulated DEGs in M217 (*P*-value < 0.05). However, this result may be influenced by incomplete KEGG pathway annotations for *G. huxleyi*. In contrast, the down-regulated DEGs in M217 are associated with 11 enriched KEGG pathways, all with *P*-values < 0.05, as shown in Fig. 6. Notably, two pathways, Steroid biosynthesis (ehx00100) and AGE-RAGE signaling pathway in diabetic complications (ehx04933), are particularly enhanced, with an enrichment factor exceeding 50%. These pathways likely reflect stress responses in the CCMP1516 strain.

**Fig. 7.**
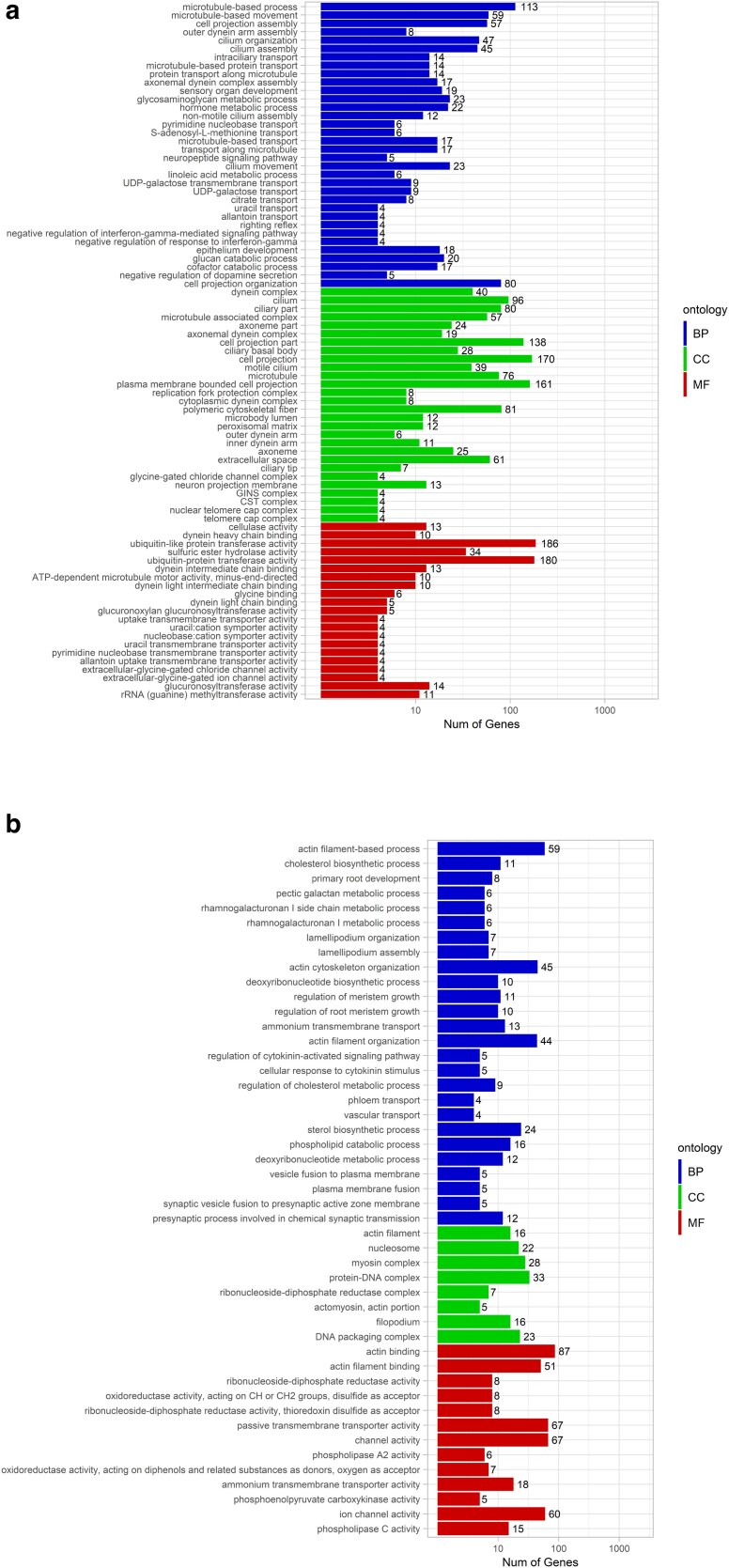
Top enriched GO terms (*P* < 0.001) of genes up-regulated (a) and down-regulated (b) in strain M217 vs CCMP1516. The numbers of DEGs annotated with each GO term are shown above the bars.

Additionally, the GO enrichment analysis revealed over-representation of certain GO terms for both up-regulated and down-regulated genes (Fig. 7 and Supplementary Fig. 8). Among the up-regulated genes in M217, GO:0030286 (dynein complex, CC) is the most significantly enriched category. Forty out of 68 genes annotated with GO:0030286 are found to be up-regulated, with a remarkably low *P*-value of approximately 1.6 × 10^−15^. Dynein, a family of cytoskeletal motor proteins responsible for movement along microtubules, plays a key role in intracellular transport. Previous studies show the dynein regulatory complex, especially in cilia-driven flow, is crucial for controlling otolith biomineralization in vertebrates. The over-representation of DEGs associated with the dynein complex and cilia suggests a potential biological mechanism for the phenotypic differences observed in coccolith calcification between the M217 and CCMP1516 strains. Specifically, the non-calcifying nature of CCMP1516 being associated with epigenetic changes resulting in the under-expression of genes linked to dynein complex, cilia, and microtubule-based processes.

For the down-regulated DEGs in M217, the most significantly over-represented GO terms are related to actin filament processes, including actin filament (CC), actin binding (MF), and actin filament-based processes (BP). This is interesting given the role actin plays during biomineralization in vesicle transport, coccolith vesicle morphology, and coccolith secretion (Durak et al. 2017). The down-regulation of actin filament processes in calcifying M217 may reflect functional prioritization and a shift away from its role in phagocytosis, endocytosis, and motility favoring stable actin scaffolding that supports efficient coccolith biomineralization.

### Correlations between differential methylations and gene expressions

The most common approaches for associating DNA methylation with gene expression changes involve identifying differentially methylated cytosine sites (DMCs) and differentially methylated regions (DMRs) in the genome, and linking them to nearby genes (Bock 2012). However, studies have often found weak correlations between hyper- or hypo-methylated DMRs and differential gene expression (VanderKraats et al. 2013; Wagner et al. 2014; Schultz et al. 2015; Schlosberg et al. 2017). Bayesian linear regression models are generally more informative when relating gene expression changes to DMRs and their methylation levels in specific genomic regions (Tang et al. 2017).

Our analysis using Bayesian linear regression models indicate that the relationship between DNA methylation changes and nearby gene expression in *G. huxleyi* is complex. To uncover connections between methylation and gene expression in *G. huxleyi*, the association between differentially methylated genes (DMGs) and differentially methylated promoters (DMPs) and differentially methylated genes (DEGs) were probed. Table 2 shows the numbers of *G. huxleyi* genes classified DEGs, as well as DMGs and DMPs. A Chi-square test revealed a significant association between DMGs and DEGs (*P* = 4.7e−6). In contrast, no significant association was observed between DMPs and DEGs (Table 2b, *P* = 0.135), suggesting that differential methylation within gene regions plays a more prominent role in regulating gene expression, which is consistent with results in other eukaryotes (Keller et al. 2016; Shahzad et al. 2025).

**Table 2.** Differentially methylated genes and promoter regions are more likely to be differentially expressed than those that are not methylated. Number of genes categorized as differentially methylated and/or differentially expressed, including both gene bodies and promoter regions.

	DMGs	Non-DMGs	DMPs	Non-DMPs
DEGs	1388	11,323	1612	11,099
Non-DEGs	2392	22,978	3081	22,289

Supplementary Fig. 12 illustrates the log fold changes in gene expression of DMGs (M217 vs CCMP1516) alongside the corresponding changes in methylation levels of overlapping differentially methylated regions (DMRs). The plot underscores the complexity of the relationship between differential gene expression and DNA methylation in *G. huxleyi*.

Differentially methylated and expressed genes (DMEGs) are defined as the intersection of DEGs and DMGs—genes that are differentially expressed between M217 and CCMP1516 and overlap with at least one DMR. A total of 1,701 DMEGs were identified, of which 873 were up-regulated in M217 and 828 were down-regulated. Enrichment analysis of the 873 up-regulated DMEGs shows “cilium” (GO:0005929) as the most enriched (*P* = 7.3e−6), cellular component with “microtubule-based movement” (GO:0007018, biological process) as the most enriched biological process (*P* = 4.2e−5).

Our analysis revealed notable differences in CpG methylation levels and gene expression. DEGs were associated with a higher number of differentially methylated cytosines (DMCs) and differentially methylated regions (DMRs) than non-DEGs (Supplementary Fig. 11). In the CpG context, DEGs had an average of 1.859 DMCs per 1,000 bp in promoter regions, compared to 1.707 for non-DEGs. Similarly, within gene regions, DEGs exhibited an average of 2.161 DMCs per 1,000 bp, compared to 1.943 in non-DEGs. Moreover, a greater proportion of DEGs overlapped with DMRs in both promoter and gene regions. Specifically, 7.4% of DEGs overlapped with at least one DMR in the CpG context, compared to 6.1% of non-DEGs. In promoter regions, 7.7% of DEGs overlapped with DMRs, compared to 7.2% of non-DEGs.

**Fig. 8.**
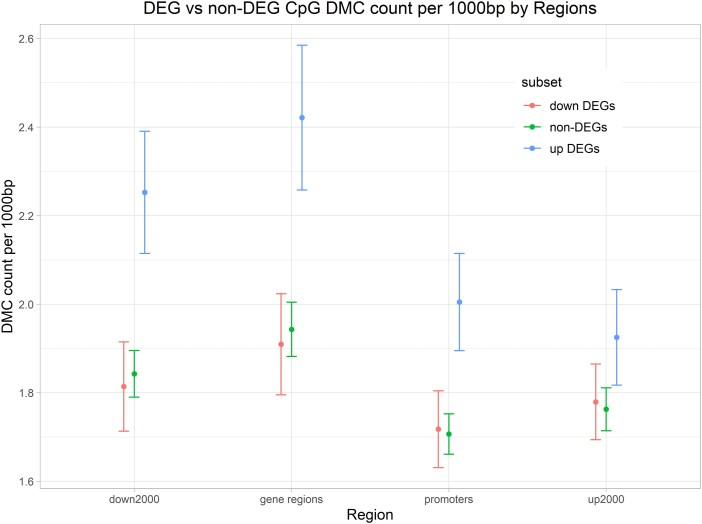
Differentially expressed genes (DEGs) exhibit distinct patterns of DNA methylation changes compared to non-DEGs. DEGs up-regulated in M217 are associated with higher counts of CpG differentially methylated cytosines (DMCs) counts per 1,000 bp, particularly in gene regions and promoters, whereas down-regulated DEGs and non-DEGs show lower counts. Error bars indicate the 99% confidence interval around the mean.

When comparing up-regulated and down-regulated DEGs in M217 relative to CCMP1516, we found that up-regulated DEGs had contained significantly more CpG DMCs. In promoter regions, up-regulated DEGs averaged 2.005 DMCs per 1,000 bp, compared to 1.718 for down-regulated DEGs. Within gene regions, up-regulated DEGs averaged 2.42 DMCs per 1,000 bp, compared to 1.91 for down-regulated DEGs. These differences, illustrated in Fig. 8, support the hypothesis that DNA methylation may be associated with the regulation of genes overexpressed in M217, particularly those involved in calcification processes.

CpG DMCs were further classified as hypo-methylated (lower methylation in M217) or hyper-methylated (higher methylation in M217). In promoter regions, up-regulated DEGs contained significantly more hypo-methylated DMCs (1.211 per 1,000 bp) than down-regulated DEGs (1.041 per 1,000 bp). They also exhibited more hyper-methylated DMCs (0.794 per 1,000 bp) compared to down-regulated DEGs (0.677 per 1,000 bp). Likewise, within gene regions, up-regulated DEGs averaged 1.472 hypo-DMCs and 0.949 hyper-DMCs per 1,000 bp, compared to 1.169 hypo-DMCs and 0.740 hyper-DMCs for down-regulated DEGs.

These findings suggest a strong correlation between CpG methylation changes and variations in gene expression, highlighting the role of methylation in regulating transcription. Nevertheless, the relationship is complex.

In the CHG context, the relationship between methylation and gene expression is less well defined. The average number of DMCs per 1,000 bp in promoter regions (1.08 for DEGs vs 1.09 for non-DEGs) and in gene regions (1.22 for DEGs vs 1.21 for non-DEGs) of differentially expressed genes is no different than that of those exhibiting similar expression. However, up-regulated DEGs exhibited more CHG DMCs in both promoters (1.148 per 1,000 bp) and gene regions (1.317 per 1,000 bp), compared to down-regulated DEGs (1.012 and 1.134 per 1,000 bp, respectively). The predominance of hypermethylated CHG DMCs in M217 reflects its significantly higher overall CHG methylation levels. These findings suggest that CHG DMCs may influence gene expressions differently from CpG DMCs.

### GLM modeling of methylation effects on gene expression

To quantitatively assess the effects of differential methylation on gene expression, we constructed a generalized linear model (GLM) (Section 5.4). The model estimated coefficients $\beta_{+}$ and $\beta_{-}$, representing the effects of hyper-methylated (higher in M217) and hypo-methylated (lower in M217) DMCs, respectively, on the logarithm of gene expression levels. Non-zero coefficients with high confidence reject the null hypothesis that DMCs are unrelated to gene expression.

**Fig. 9.**
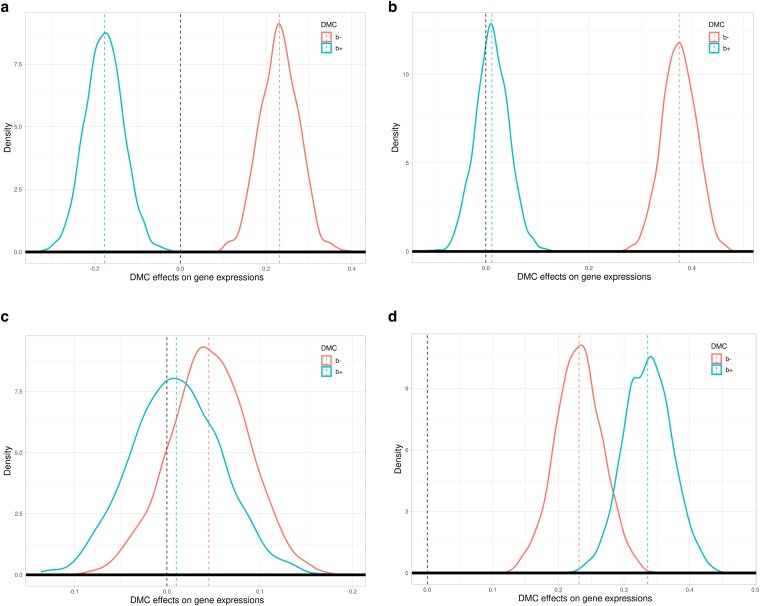
Density plots of estimated distributions of $b_{+}$, and $b_{-}$, where $b_{+}$ represents the effects of hyper-methylated DMCs (in M217) and $b_{-}$ represents effects of hypo-methylated DMCs. Hypo-methylated CpG DMCs in gene bodies (a) and promoter regions (b) correlate with higher gene expression in M217, while hyper-methylation in gene bodies correlates to lower expression in M217 (a). Both hyper-methylated and hypo-methylated CHG DMCs (c and d) appear to have a positive but more varied correlation with elevated gene expression in the calcifying M217.

In the CpG context, both $\beta_{+}$ and $\beta_{-}$ were significantly different from zero for DMCs in gene regions, with mean values of $\beta_{+}=-0.18$ and mean $\beta_{-}=0.23$ (standard deviation = 0.04). The signs of these coefficients are consistent with prior findings: hyper-methylation tends to suppress, while hypo-methylation tends to enhance gene expression. Figure 9 shows the density plots of the estimated posterior distribution of $\beta_{+}$ and $\beta_{-}$. In promoter regions, the mean values were $\beta_{+}=0.01$ and mean $\beta_{-}=0.38$ (standard deviation = 0.03), indicating that hyper-methylation in promoters had minimal impact on gene expression, whereas hypo-methylation was strongly correlated with increased expression. These results suggest that elevated DNA methylation levels in CCMP1516 may silence specific genes, contributing to its phenotypic changes and loss of calcification ability.

In the CHG context, the values of $\beta_{+}$ and $\beta_{-}$ in gene regions were close to zero ($\beta_{+}=0.01$, std = 0.05; $\beta_{-}=0.04$, std = 0.04), supporting the null hypothesis that CHG DMCs in gene regions have minimal impact on gene expression. However, in promoter regions, both $\beta_{+}=0.33$ (std = 0.04) and $\beta_{-}=0.23$ (std = 0.04) were significantly different from zero, suggesting that both hyper- and hypo-methylated CHG DMCs in promoters contribute to enhanced gene expression in M217.

## Discussion

Because of the ecological and biogeochemical importance of coccolithophores, considerable research has focused on elucidating the mechanisms of calcification. Electron microscopy and time-lapse imaging have revealed cellular details of biomineralization known to occur in a specialized organelle designated as the coccolith vesicle (CV). The CV originates from the Golgi apparatus and is closely associated with the endomembrane system. A reticular body juxtaposed to the CV facilitates vesicle transport between the Golgi apparatus and the CV, regulating the supply of ions required for coccolith formation. The nucleation and growth of calcium carbonate crystalline elements occur on an organic baseplate and are thought to be largely controlled by polysaccharides (Gal et al. 2016; Taylor et al. 2017). Upon maturation, the newly formed coccolith migrates to the plasma membrane where it is extruded via exocytosis. While these cellular and structural details are well established, the mechanisms by which biomolecules —including proteins, polysaccharides, proteoglycans, and proteolipids—regulate coccolith crystal nucleation and growth remain unclear. The epigenetic and RNA-seq data presented here, comparing closely related calcifying and non-calcifying *G. huxleyi* cell lines, provide new insight into the biomolecules and potential processes involved in biomineralization in this important species.

### Dynein

The most significantly enriched GO term for the differentially expressed genes was the dynein complex (GO:0030286), a multi-subunit motor protein that uses ATP hydrolysis to move along microtubules facilitating intracellular transport, mitosis, and ciliary/flagellar movement. The up-regulation of these proteins in calcifying vs non-calcifying cultures of *G. huxleyi* is consistent with a potential indirect role for cytoskeletal motor activity during biomineralization. In coordination with kinesin and other cytoskeletal elements, dynein could contribute to the transport of Golgi-derived vesicles carrying Ca^2+^, bicarbonate, and polysaccharides to the coccolith vesicle, and/or to the positioning and movement of the coccolith vesicle prior to extrusion at the plasma membrane. It is also possible that dynein participates in the fibrillar machinery implicated in shaping the coccolith vesicle and organizing biomineral deposition. When treated with cytoskeletal inhibitors, colchicine and cytochalasin B, *G. huxleyi* produces malformed coccoliths, and it is now widely accepted that microtubules and actin microfilaments contribute to a dynamic fibrillar structure essential not only for shaping the vesicle and the developing coccolith, but also for generating the force required to expel coccoliths to the cell surface (Langer et al. 2010; Walsh et al. 2018). Dynein may function within this broader cytoskeletal network, although its specific role remains to be determined.

### Phosphate

Relative to the non-calcifying CCMP1516, M217 shows a marked increase in expression of a phosphate transporter (PID 61414; ∼870-fold higher *P* = 0), an acid phosphatase (PID 228712; ∼55-fold), an alkaline phosphatase (PID 218706; ∼4.5-fold), and multiple inorganic pyrophosphatases (PIDs 350751, 336828, 44801; 7 to 13-fold). This aligns with previous transcriptomic and proteomic studies showing coordinated up-regulation of these and other phosphorus acquisition pathways in calcifying strains and under calcification favoring conditions (Rokitta et al. 2014, 2016). Calcification in *G. huxleyi, however,* is frequently associated with oligotrophic, low-phosphate environments (Tyrrell and Taylor 1996; Paasche 1998). Results herein suggest perhaps that calcification in *G. huxleyi* relies on enhanced access to both external dissolved organic phosphorus and internal phosphorus recycling. Consistent with this interpretation, previous studies describe intracellular compartments in coccolithophores enriched in calcium and phosphate (Sviben et al. 2016; Kadan et al. 2020; Peled-Zehavi and Gal 2021); although not unequivocally linked to biomineralization, these structures may reflect cellular strategies for transient ion sequestration or homeostasis under conditions of high elemental flux. Elevated pyrophosphatase expression, could point to increased PPi turnover associated with high biosynthetic and energetic fluxes, such as membrane remodeling and biogenesis, ATP cycling, intracellular pH and ion balance, and vesicle trafficking required for coccolith formation. Hence, the success of calcifying strains in low-phosphate environments may depend upon high-affinity uptake and rapid recycling rather than intrinsic phosphorus independence, with the observed induction of phosphate transporters, alkaline phosphatase, and related pathways reflecting adaptive mechanisms that sustain calcification.

The pronounced differential expression of the phosphate transporter also appears to be associated with DNA methylation patterns, as the gene overlaps two DMRs located 18 bp upstream and 72 bp downstream of its TSS. Both DMRs were hypo-methylated in M217 compared to CCMP1516, with average methylation differences of 28.4 and 39%, respectively. This correlation suggests that higher methylation levels in CCMP1516 may be linked to reduced expression of the transporter, although a causal relationship cannot be inferred from these data. Notably, one of the pyrophosphatase (PID 44801) was upregulated in the calcifying strain M217 but also exhibited reduced methylation (DMRs average ∼−17.5%) compared to the non-calcifying CCMP1516, further supporting an association between phosphate-related gene regulation and the calcifying phenotype.

### Cilia and flagella-related protein

It is noteworthy that genes related to cilia and flagella are enriched in calcifying M217 (Supplementary Table 4), despite the absence of these structures in the diploid phase of the *G. huxleyi*. These findings raise the possibility that such genes may have additional, non-canonical roles potentially unrelated to motility, including functions intracellular transport, cytoskeletal dynamics, or stress responses. Cilia- and flagella-associated proteins 58, 61, and 70 (PIDs 66449, 122188, and 194332) are notably upregulated more than 50-fold, yet their functional annotation remains unclear. One hypothesis is that these proteins represent retained components from the flagellated haploid phase that continued to be expressed in the diploid phase, potentially with altered or residual functions. Alternatively, they may represent broader conserved proteins that, while essential for cilia/flagella formation in other organisms, have been co-opted for different cellular processes in *G. huxleyi*.

The strong upregulation of a tubulin-tyrosine ligase family protein (PID 438199), multiple dynein-associated proteins (PIDs 198139, 229544, 20030, 63754, 117244, 211505), and myosin (PID 219951) may be consistent with increased cytoskeletal dynamics, potentially associated with cell shape changes, intracellular transport, and cellular reorganization during coccolith formation. These observations align with (Skeffington et al. 2023), who reported coccolith-associated myosins and kinesins and an expanded complement of myosin head–containing proteins in *G. huxleyi.* Calcification and coccolithogenesis involve extensive membrane remodeling—including vesicle formation, fusion, and the exocytosis of coccoliths—processes that are theoretically compatible with the presence of a perforin domain-containing protein (PID 106349), which is upregulated four-fold and, in plants, has been linked with development and stress responses via membrane pore formation (Yu et al. 2020).

Nevertheless, direct evidence linking these cilia- and flagella-related proteins to calcification and coccolithogenesis is currently lacking, and these interpretations should be regarded as speculative, warranting further investigation.

### Carbonic anhydrase

Carbonic anhydrase (CA) is widely implicaed in biomineralization across diverse marine organisms, including the formation of carbonate structures in mollusks, echinoderms, corals, and phytoplankton, due to its role in regulating inorganic carbon chemistry through the reversible reaction, CO_2_ + H_2_O⇔H + +HCO_3_, thereby regulating the availability of bicarbonate and carbonate ions. Like other organisms, *G. huxleyi* encodes multiple CA isozymes that are thought to support a range of cellular processes beyond calcification, including carbon fixation, ion regulation, and other general metabolic functions. Several studies have specifically implicated CA activity in coccolithophore calcification: (Von Dassow et al. 2009) identified a β-BA that was specific to the calcifying (1N) life-cycle stage, (Mackinder et al. 2011) examined CA expression patterns in relation to calcification, and more recently (Skeffington et al. 2023) detected β-CAs across multiple proteomic datasets associated with coccolith formation.

Although α-CAs and β-CAs are most commonly linked to biomineralization processes (Chang et al. 2012; Voigt et al. 2014; Del Prete et al. 2017), our data indicate CA isoform regulation in *G. huxleyi* is more complex. Among the 15 CA isozymes encoded in *G. huxleyi*, one δ-CAs (PID 195575) is expressed at markedly higher levels (∼130-fold; *P* = 0) in calcifying M217 compared to CCMP1516, a difference that is accompanied by reduced methylation (23%) 1,319 bp downstream of the TSS. In contrast, one α-CAs (PID 437452) shows substantially lower expression (>10-fold) in M217. These opposing expression patterns suggest transitions between calcifying and non-calcifying states in *G. huxleyi* are associated with selective regulation of specific CA isoforms, rather than a uniform upregulation CA activity. The elevated expression of the δ-CA may be consistent with increased cellular demand for bicarbonate and CO_2_ under calcifying conditions, whereas suppression of the α-CA could reflect shifts in carbon processing pathways that accompany calcification. However, as with the cilia- and flagella-associated proteins discussed above, these interpretations are based on correlative expression and epigenetic data. Determining the functional relevance of individual CA isozymes will require direct investigation of their enzymatic activity, intracellular location, and life-cycle specific roles.

### Other notable proteins

Numerous proteins previously associated with biomineralization-related processes show elevated expression in M217 compared with CCMP1516. These include a diverse set of calcium ion-binding proteins (PIDs 219537, 207498, 231735, 424741, 422325, 256219, 438601, 98874, 459830, 313977, 112592, 121139), calmodulin (PID 219537), calcium-dependent kinases (PID 242080, 207498), and multiple EF-hand domain-containing proteins. In other systems, these protein families participate in calcium sensing, buffering, and calcium dependent signaling, however, their specific roles in *G. huxleyi* calcification remain unresolved. The “glutamic acid-proline-alanine” glycoprotein (GPA) previously linked to *G. huxleyi* coccoliths through biochemical and immunolocalization studies (Corstjens et al. 1998) is notably absent among the differentially expressed genes.

Calcium is a well-established second messenger in eukaryotic cells, and changes in the expression of calcium-binding and calcium responsive proteins may align with altered signaling dynamics under calcifying conditions. Rather than indicating direct control of calcification, the coordinated upregulation of these genes likely reflects broader cellular adjustments in calcium homeostasis that accompany coccolith formation. In this context, calcium may function not only as a structural component of coccoliths but also as part of a signaling network associated with the calcifying state.

Ankyrin repeat containing proteins, which serve as protein-protein interaction domains in many cellular contexts, were also enriched among the upregulated genes in M217. Several ankyrin-containing proteins (PIDs 232825, 107830, 120189, 114677, 220893, 463290, 104578) exhibit significant significantly higher expression in the calcifying strain, consistent with recent reports identifying ankyrin repeat proteins associated with the coccosphere in *G. huxleyi* (Skeffington et al. 2023). In other organisms, these proteins often act as molecular scaffolds involved in transcriptional regulation, ion transport, and enzymatic organization. It is therefore plausible that ankyrin repeat-containing proteins contribute to cellular organization or matrix-associated processes, during calcification, including the structuring of the organic matrix that underpins coccolith formation in *G. huxleyi* (Walsh et al. 2018). Nonetheless, direct evidence linking these proteins to biomineralization is currently lacking and their roles remain to be experimentally determined.

## Conclusions

This study reveals how epigenetic mechanisms, including differential DNA methylation, may serve as important regulators of gene expression in transporters, enzymes, and structural proteins critical to biomineralization in *G. huxleyi*, underscoring the intricate relationship between environmental cues and internal gene regulatory networks. The identification of specific motor proteins, signaling molecules, and ion regulatory factors not previously implicated in coccolith formation expands the molecular framework through which *G. huxleyi* orchestrates calcification, providing new targets for functional investigation and ecological modeling.

Determining the structure, spatial-temporal localization, and—most importantly—the function of mineralization-associated proteins remains a significant challenge, particularly in the absence of a genetic transformation system and the inability to knock out or overexpress specific proteins in *G. huxleyi*. While CRISPR technologies hold great promise, the development of reliable delivery systems is urgently needed. In the meantime, genomic, epigenetic, and transcriptomic studies such as those presented here offer powerful tools for gaining functional insights into biomineralization pathways and their integration with the physiological and ecological drivers that regulate this process.

## Supplementary Material

jkag076_Supplementary_Data

## Data Availability

The WGBS and RNA_seq sequencing data have been deposited in the NCBI Sequence Read Archive (SRA) (BioProject: PRJNA894788). The example code used in this study, along with the output data for differentially methylated cytosines (DMCs), differentially methylated regions (DMRs), differentially expressed genes (DEGs), and differentially methylated genes (DMGs) and promoters (DMPs), is available at: https://github.com/xiaoyu12/methylation/. Supplemental material available at G3 online.
