# Supplementary material for: Integration of DNA methylation and gene expression analysis in Gephyrocapsa huxleyi provides insight into genes related to calcification
Source: G3 (Bethesda). 2026 May 20;16(7):jkag076. doi: 10.1093/g3journal/jkag076 (PMC13334186; doi:10.1093/g3journal/jkag076)
Supplement: jkag076_Supplementary_Data [file jkag076_supplementary_data.zip › Supplemental_Material_G3-2026-406518.pdf]

Supplementary Table 1. Statistics of the six WGBS libraries, with three biologically replicated samples of strain CCMP1516 and M217.

Sample Name	Clean Reads	Clean bases	Read length(bp)	Q20 (%)	GC (%)
**CCMP1516A**	40,161,824	6,024,273,600	150	97.37%	31.33%
**CCMP1516B**	41,579,220	6,236,883,000	150	96.92%	34.48%
**CCMP1516C**	38,142,830	5,721,424,500	150	98.81%	33.74%
**M217A**	40,114,082	6,017,112,300	150	97.21%	34.53%
**M217B**	41,574,136	6,236,120,400	150	96.82%	34.80%
**M217C**	41,648,326	6,247,248,900	150	96.86%	34.25%

Supplementary Table 2. Statistics of the six RNA-Seq libraries, with three biologically replicated samples of strain CCMP1516 and M217.

Sample Name	Clean Reads	Clean bases	Read length (bp)	Q20(%)	GC (%)
**CCMP1516A**	49,237,758	4,923,775,800	100	95.24%	66.11%
**CCMP1516B**	54,073,336	5,407,333,600	100	94.92%	67.43%
**CCMP1516C**	53,909,048	5,390,904,800	100	94.80%	67.72%
**M217A**	49,297,440	4,929,744,000	100	95.04%	67.44%
**M217B**	51,206,106	5,120,610,600	100	94.86%	67.51%
**M217C**	51,710,230	5,171,023,000	100	94.87%	67.49%

Supplementary Table 3. Mapping statistics of RNA-Seq datasets to the *G. huxleyi* reference genome.

Sample Name	% Uniquely mapped	% mapped to multiple loci	% unmapped
**CCMP1516A**	56.25%	25.24%	17.71%
**CCMP1516B**	62.00%	30.53%	6.14%
**CCMP1516C**	61.70%	30.54%	5.96%
**M217A**	58.56%	28.50%	11.90%
**M217B**	57.38%	28.77%	13.13%
**M217C**	57.42%	28.75%	12.39%

Supplementary Table 4. List of genes in DEMGs with "cilium" (GO:0005929: CC) annotation

	log2FC	Description
**106349**	1.909857	membrane attack complex perforin domain-containing
**117244**	1.950292	cytoplasmic dynein 2 heavy chain 1 isoform X1
**122188**	5.729132	cilia- and flagella-associated 61
**194332**	7.210228	cilia- and flagella-associated 70
**198139**	6.098505	Dynein heavy chain partial
**198190**	5.422113	central pair associated wd-repeat
**207330**	7.682979	dyslexia susceptibility 1 candidate gene 1 homolog

**211505**	6.698345	flagellar outer dynein arm heavy chain beta
**212369**	2.16351	hypothetical protein EMIHUDRAFT_212369
**216525**	7.940246	monoglycylase TTLL8
**219951**	7.99493	myosin heavy
**223628**	7.676854	fibrocystin-l
**229544**	5.850834	dynein intermediate chain axonemal
**230030**	6.845327	Dynein heavy chain axonemal
**243453**	7.289079	FAM179B isoform X1
**252292**	2.002519	TRAF3-interacting 1
**438199**	1.924581	Tubulin-tyrosine ligase family
**63754**	6.800667	Dynein regulatory complex 1
**66449**	7.778062	Cilia- and flagella-associated 58
**77679**	7.837322	intraflagellar transport 57 homolog

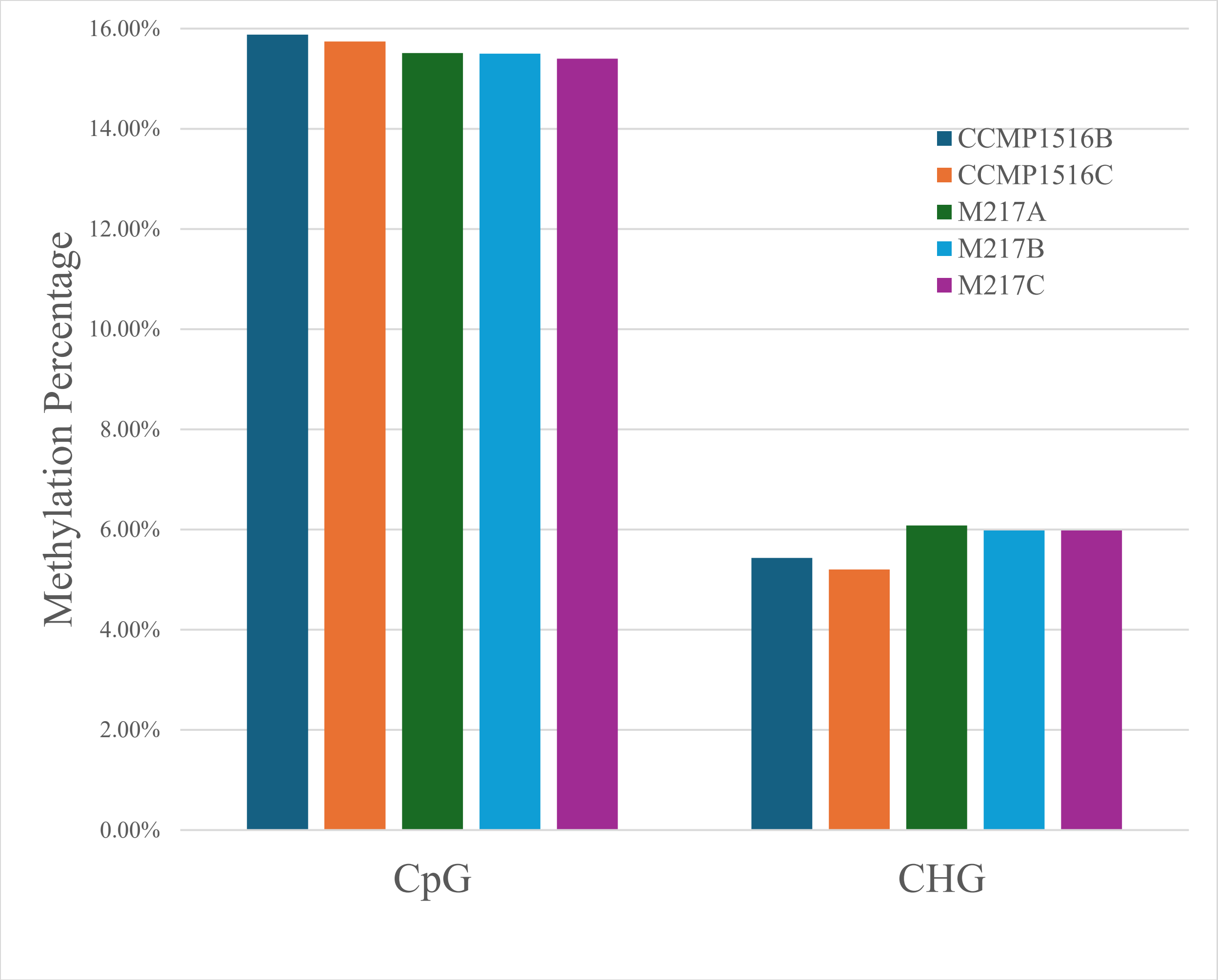

Supplementary Figure 1. Comparison of average cytosine methylation levels in the CpG and CHG contexts across the G. *huxleyi* samples. CCMP1516 exhibits significantly higher methylation levels in the CpG context and lower levels in the CHG context.

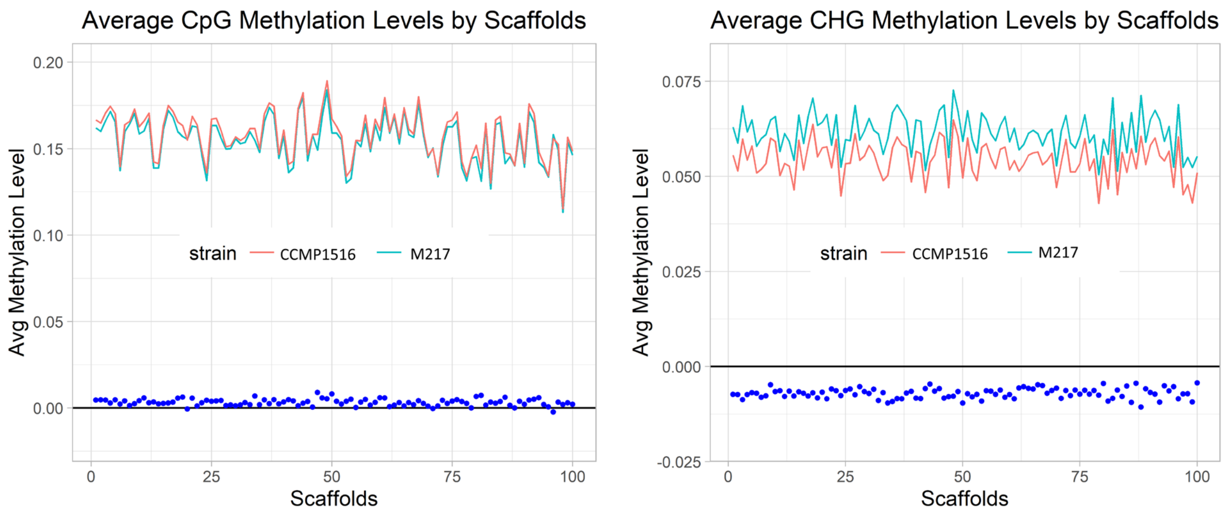

Supplementary Figure 2. Average methylation levels are consistently different across the 100 longest scaffolds of the *G. huxleyi* genome. While there is little difference in CpG methylation across the calcifying CCMP217 and the non-calcifying CCMP1516 strains, methylation is uniformly higher in M217 with respect to CHG. Blue dots indicate differences in methylation levels between CCMP1516 and CCMP217. While CpG (left) and CHG (right) methylation

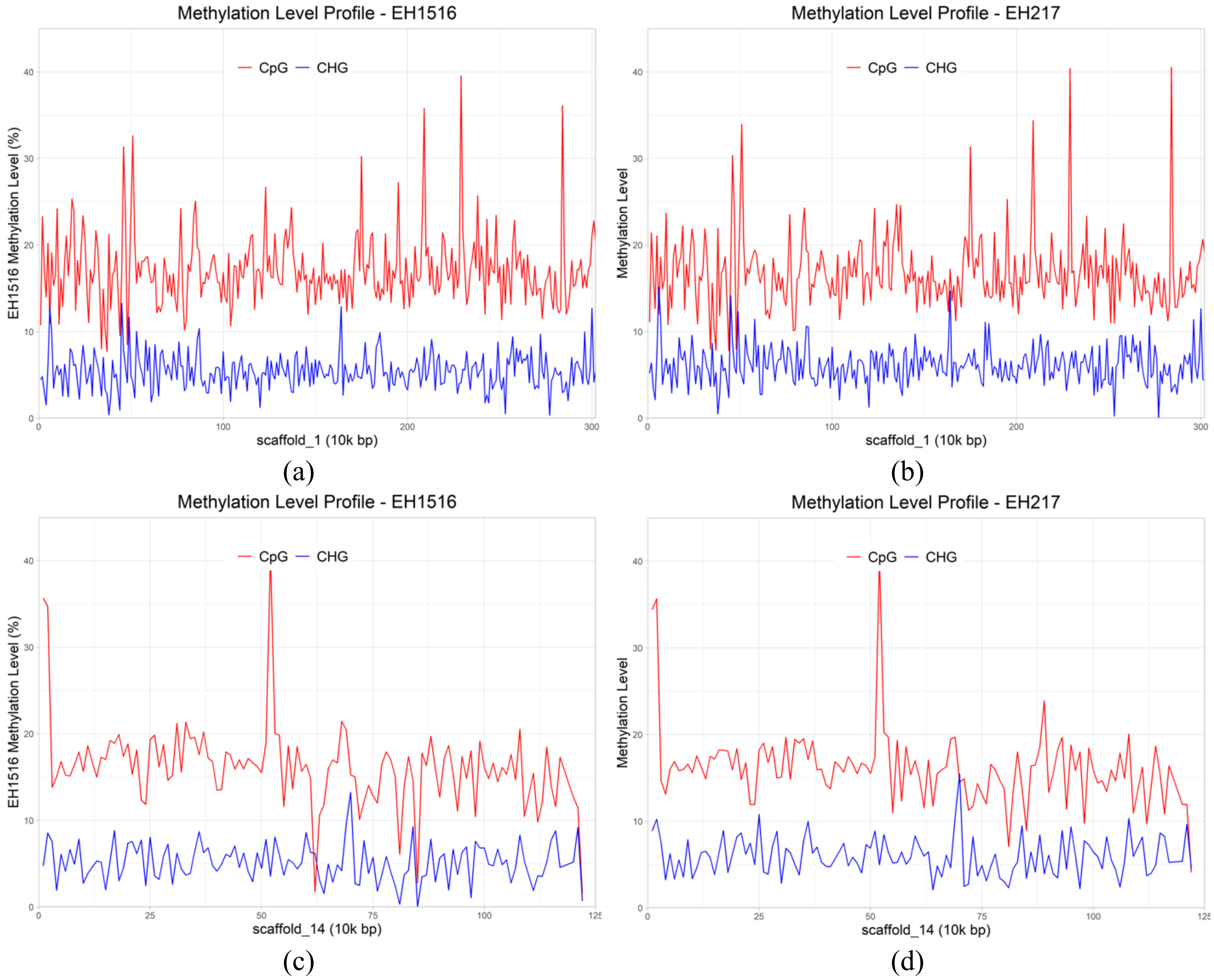

Supplementary Figure 3. Within 10-kb windows across scaffolds 1 and 14, CpG methylation levels are consistently higher than CHG level in strains CCMP1516 and M217. While the two strains show similar methylation profiles across scaffold 1 (a and b), differences emerge in scaffold 14 (c and d) where CCMP1516 exhibits more fluctuation and higher methylation peaks, particularly around the 50-kb region.

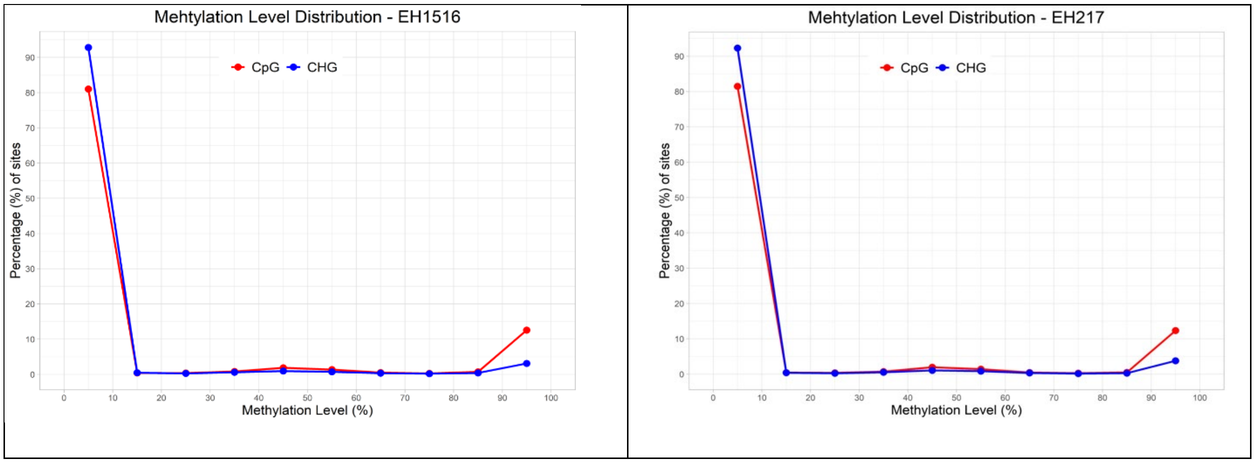

Supplementary Figure 4. Distribution of Cytosine sites according to their methylation levels under different contexts. Only cytosine sites with coverage of at least 10 were used.

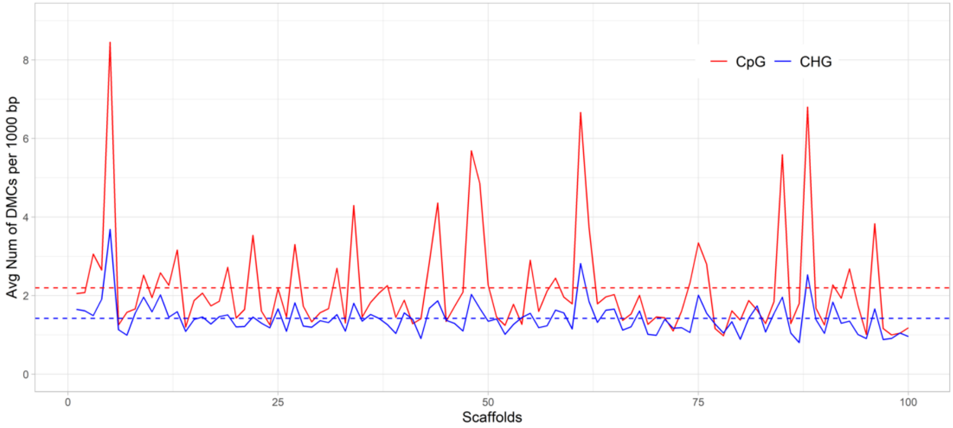

Supplementary Figure 5. Differential methylation is prevalent in the CpG context and unevenly distributed across the genome, as shown by the average rates of differentially methylated cytosines (DMCs) per 1,000 bp across the 100 longest scaffolds. Specific hot-pot scaffolds display particularly high densities of CpG DMCs, whereas CHG differential methylation is comparatively low.

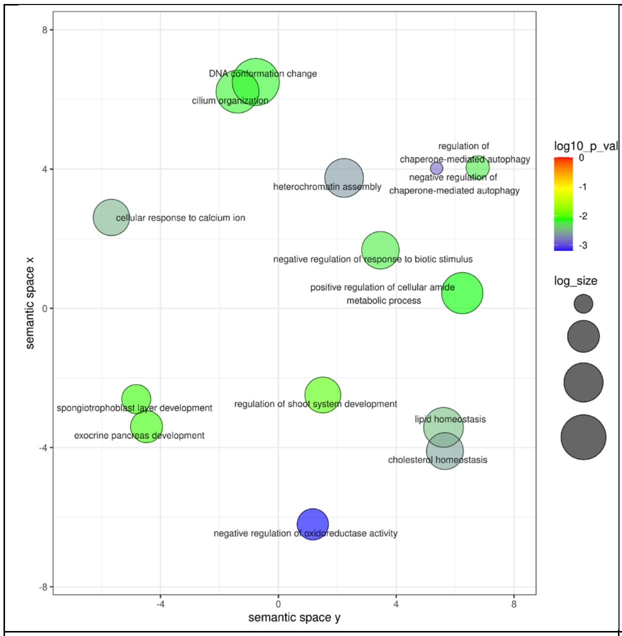

(A) DMGs enriched BP GO terms

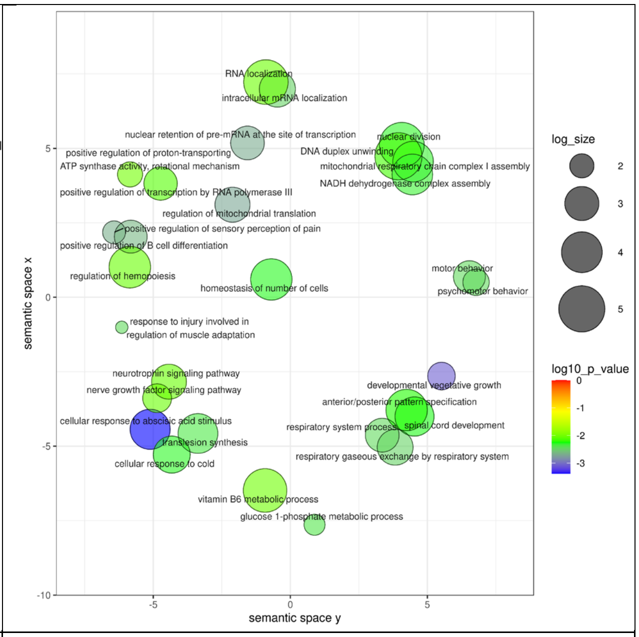

(B) DMPs enriched BP GO terms

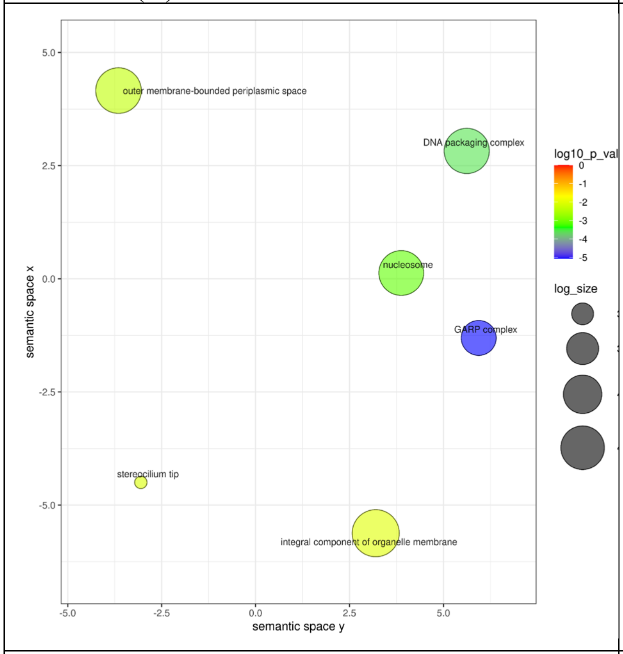

(C) DMGs enriched CC GO terms

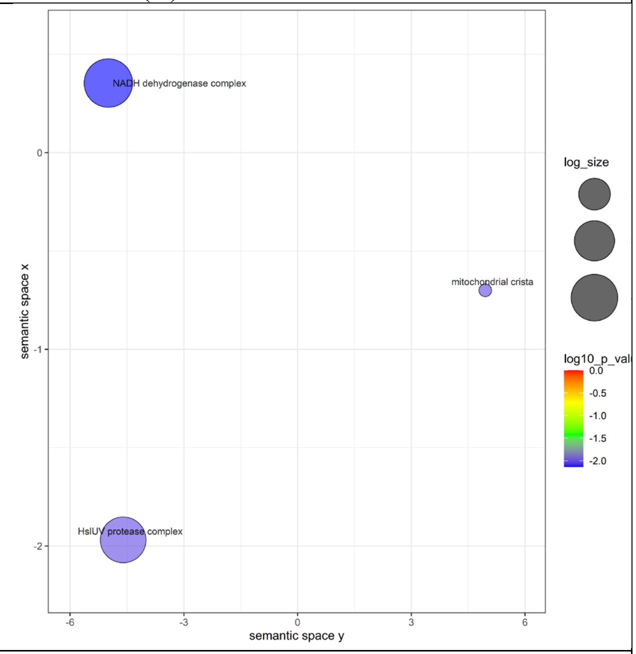

(D) DMPs enriched CC GO terms

Supplementary Figure 6. Bubble plots of enriched biological process (BP) and cellular component (CC) GO terms in DMGs and DMPs. Methylation changes in gene bodies (a, c) or promoters (b, d) influence the functional categories affected. Metabolic and regulatory processes are consistently enriched in the context of differential methylation of gene and promoter regions. DMGs are associated with general cellular, metabolic, and developmental processes, as well as responses to stimuli, whereas DMPs are enriched in terms related to specific primary metabolic pathways (nucleotide and nucleic acid metabolism), fundamental cellular machinery (protein phosphorylation, DNA replication), and specific cellular compartments such as mitochondria.

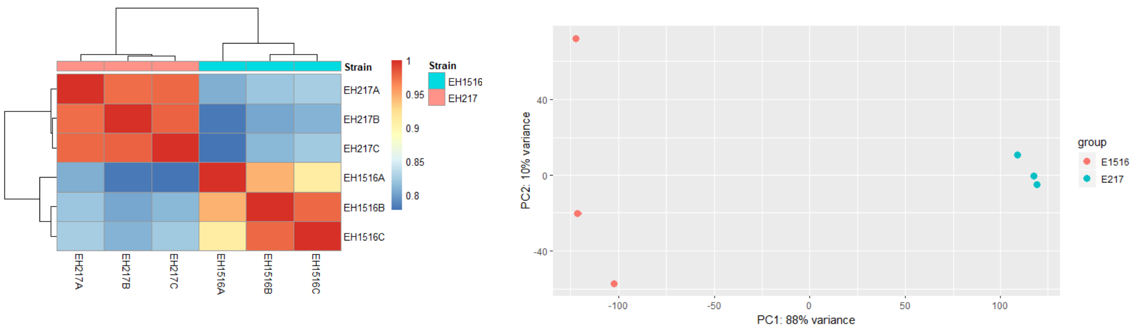

(a) (b)

Supplementary Figure 7. (a) Correlations plots and (b) PCA plots for normalized gene expression profiles of the six *G. huxleyi* samples. CCMP1516 and M217 exhibit distinct and reproducible gene expression profiles, indicating significant underlying biological differences in their transcriptional activity.

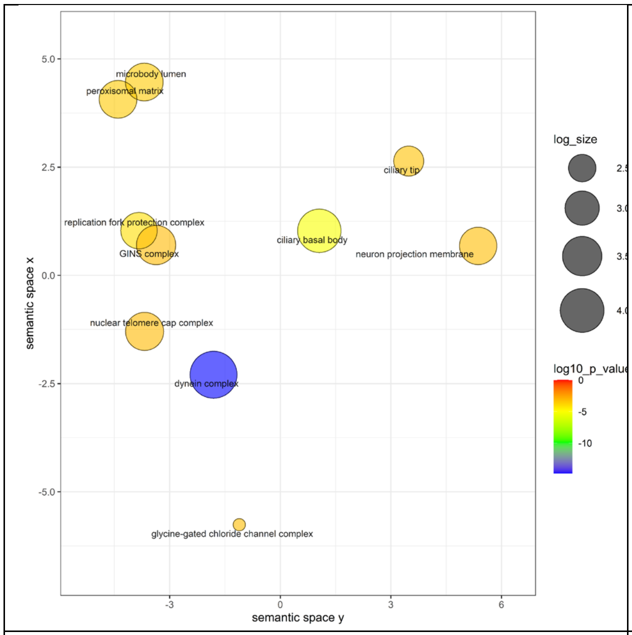

(A) CC GO terms enriched in DEGs up-regulated in M217

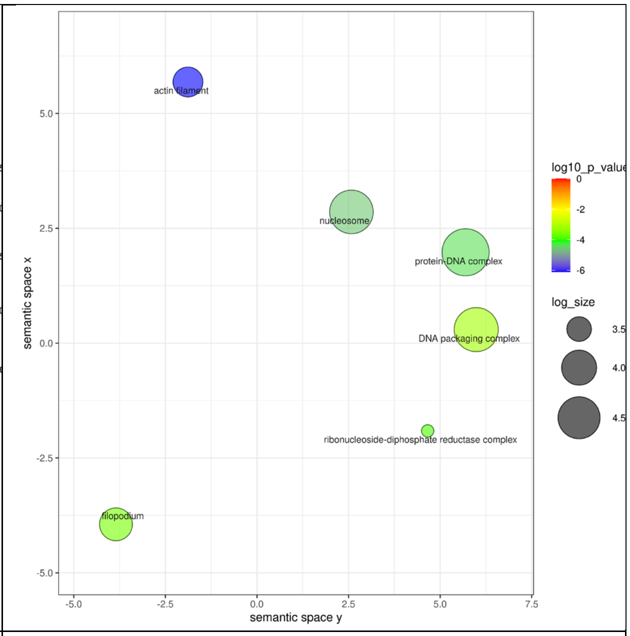

(B) CC GO terms enriched in DEGs down-regulated in M217

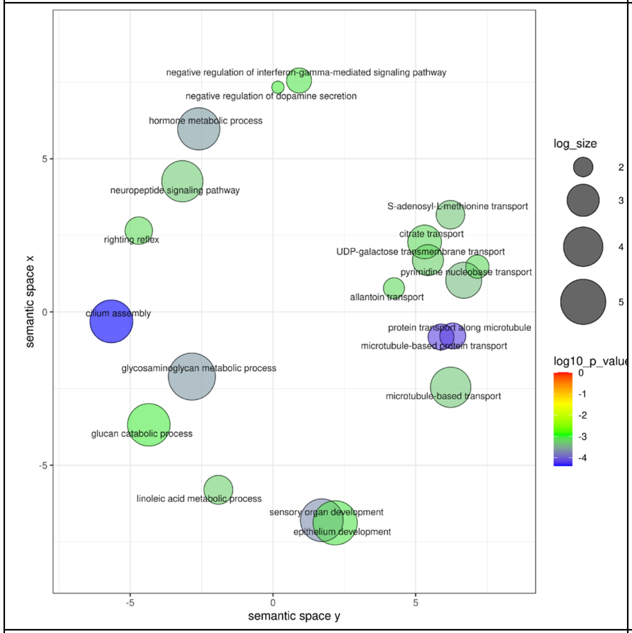

(C) BP GO terms enriched in DEGs up-regulated in EH217

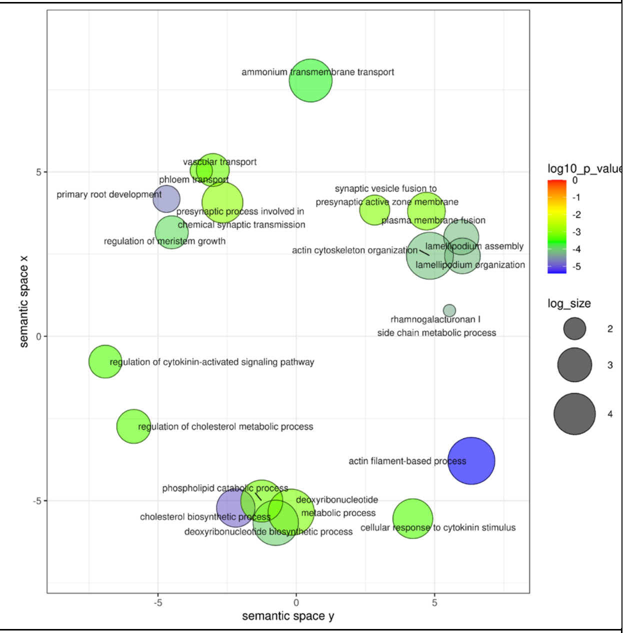

(D) BP GO terms enriched in DEGs down-regulated in EH217

Supplementary Figure 8. Bubble plots of enriched cellular component (CC) and biological process (BP) GO terms in DEGs up-regulated and down-regulated in M217 vs. CCMP1516.

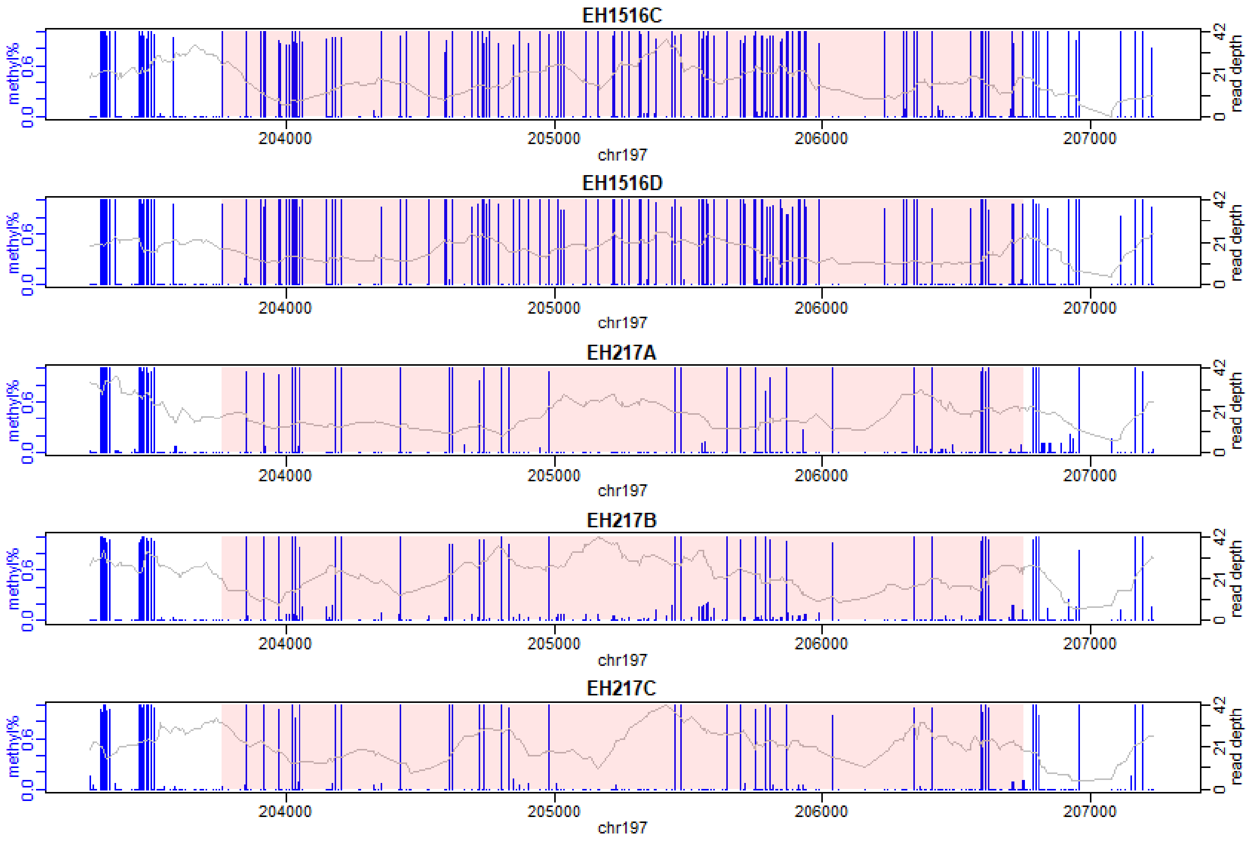

Supplementary Figure 9. An example DMR showing the differential methylation patterns between the CCMP1516 and M217 strains.

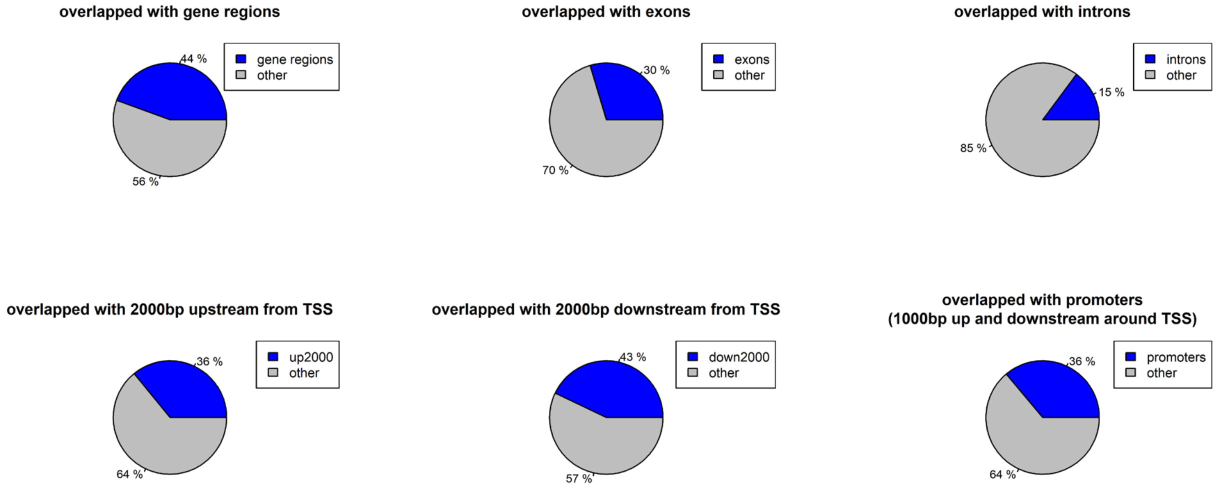

Supplementary Figure 10. The percentages of DMCs in the CHG context overlapped with various feature regions.

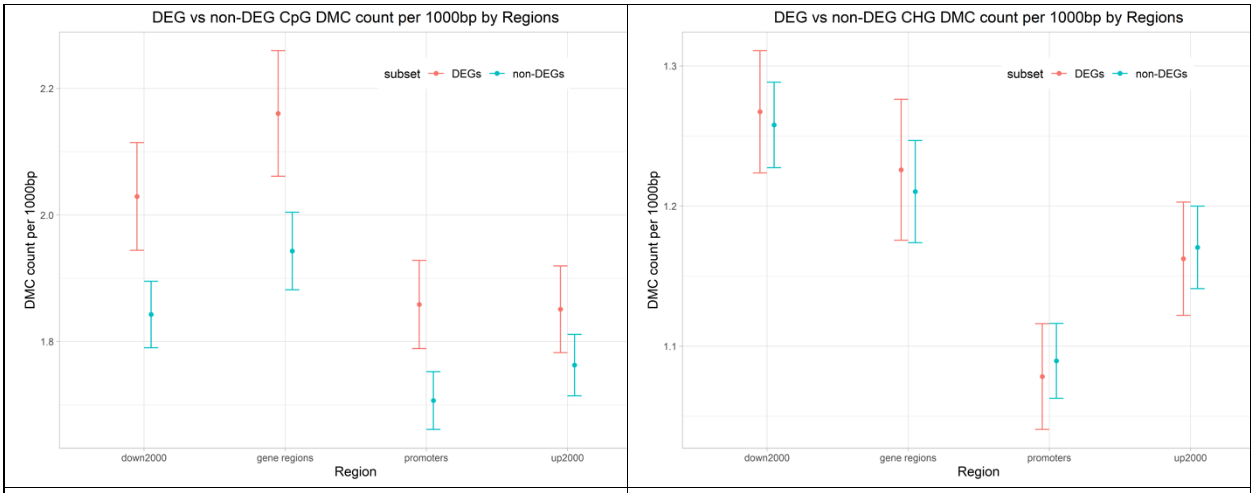

fa(a)	(b)

Supplementary Figure 11. Comparison of CpG (a) and CHG (b) DMC count per 1000 bp in different feature regions. The error bars showed the 99% confidence interval around the mean.

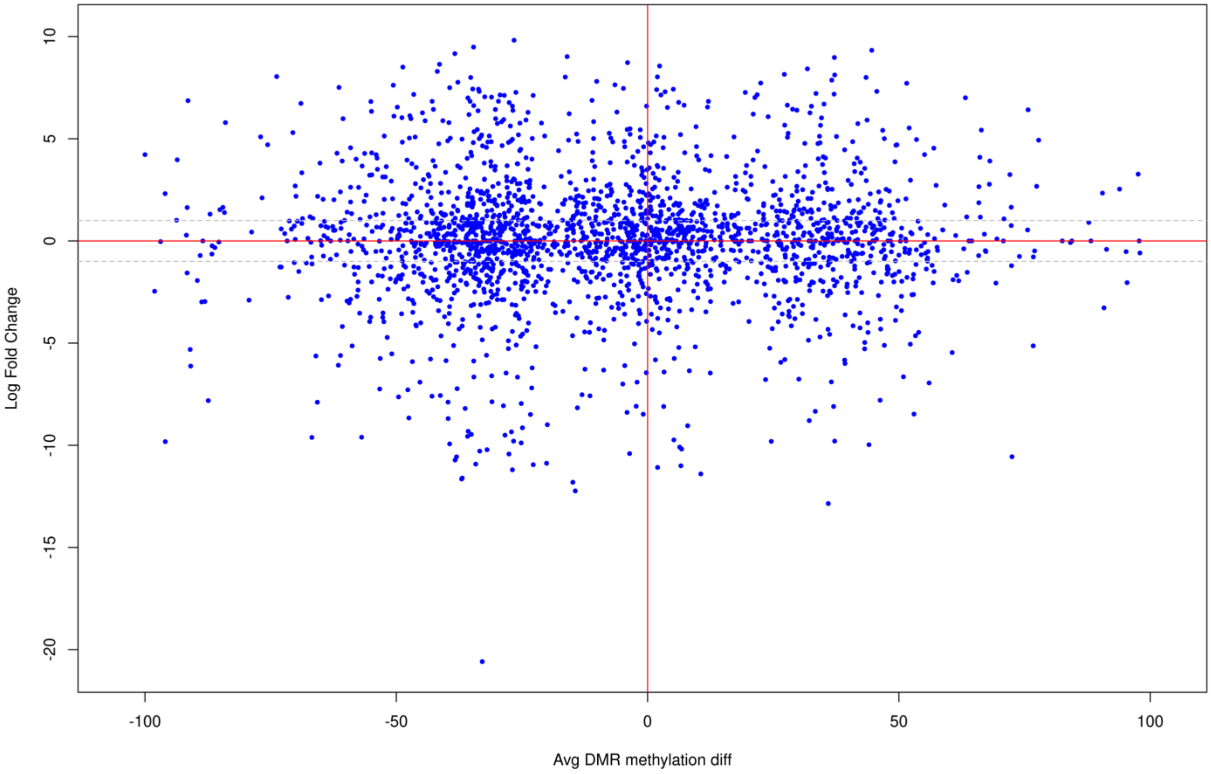

Supplementary Figure 12. Plot of gene expression Log-Fold-Change (M217 vs. CCMP1516) and average methylation differences of associated DMRs for DMGs. Each dot represents one DMG and note that a DMG may contain multiple DMRs, either hyper or hypo-methylated.
